# Psychometric Properties of a Delirium Severity Score for Older Adults and Association With Hospital and Posthospital Outcomes

**DOI:** 10.1001/jamanetworkopen.2022.6129

**Published:** 2022-03-31

**Authors:** Sarinnapha M. Vasunilashorn, Tamara G. Fong, Benjamin K. I. Helfand, Tammy T. Hshieh, Edward R. Marcantonio, Eran D. Metzger, Eva M. Schmitt, Patricia A. Tabloski, Thomas G. Travison, Yun Gou, Richard N. Jones, Sharon K. Inouye

**Affiliations:** 1 Department of Medicine, Harvard Medical School, Boston, Massachusetts; 2 Division of General Medicine, Department of Medicine, Beth Israel Deaconess Medical Center, Boston, Massachusetts; 3 Department of Epidemiology, Harvard T.H. Chan School of Public Health, Boston, Massachusetts; 4 Aging Brain Center, Marcus Institute for Aging Research, Hebrew SeniorLife, Boston, Massachusetts; 5 Department of Neurology, Beth Israel Deaconess Medical Center, Boston, Massachusetts; 6 Department of Emergency Medicine, University of Massachusetts Medical School, Worcester; 7 Department of Medicine, Brigham and Women’s Hospital, Boston, Massachusetts; 8 Department of Psychiatry, Beth Israel Deaconess Medical Center, Boston, Massachusetts; 9 Department of Nursing, William F. Connell School of Nursing at Boston College, Boston, Massachusetts; 10 Department of Psychiatry and Human Behavior, Brown University Warren Alpert Medical School, Providence, Rhode Island; 11 Department of Neurology, Brown University Warren Alpert Medical School, Providence, Rhode Island; 12 Associate Editor, *JAMA Network Open*

## Abstract

**Importance:**

The ability to rate delirium severity is key to providing optimal care for older adults, and such ratings would allow clinicians to target patients with severe delirium and monitor response to treatment, recovery time, and prognosis; assess nursing burden and staffing needs; and, ultimately, provide more appropriate patient-centered care. Current delirium severity measures have been limited in their content, gradations, and measurement characteristics.

**Objective:**

To examine the internal consistency, reliability, and validity for clinical outcomes of the DEL-S delirium severity score, a measure of delirium severity that was developed using advanced psychometric approaches, analogous to those of the Patient-Reported Outcomes Measurement Information System initiative.

**Design, Setting, and Participants:**

This prospective cohort study was conducted at a large academic medical center in Boston, Massachusetts. Adults aged 70 years or older who were admitted or transferred to medical or surgical services as either emergency or elective admissions were enrolled between October 20, 2015, and March 15, 2017, and were monitored for 1 year. Data analysis was performed from June 2020 to August 2021.

**Exposures:**

Delirium severity, measured by scores on the delirium severity score short-form (SF; 6 items, scored 0-13, with higher scores indicating more severe delirium) and long-form (LF; 17 items, scored 0-21), considered continuously and grouped into 5 categories.

**Main Outcomes and Measures:**

The primary outcomes were in-hospital outcomes, including length of stay and hospital costs, and posthospital (30, 90, and 365 days) outcomes, including death, health care costs, and rehospitalization.

**Results:**

The 352 participants had a median (IQR) age of 79.7 (74.6-85.5) years, 204 (58.0%) were women, and they were highly educated (median [IQR] duration of education, 14 [7-20] years). Patients in the highest delirium severity score SF group (scores 6-9) had a longer length of stay (13.3 vs 6.9 days; *P* for trend < .001), greater in-hospital costs ($57 700 vs $34 200), greater cumulative health care costs ($168 700 vs $106 500; *P* for trend = .01), and increased mortality at 1 year (50% vs 17%; *P* for trend = .02) compared with patients in the lowest delirium severity score SF group (score 0). Similar trends and significant findings were demonstrated for the delirium severity score LF.

**Conclusions and Relevance:**

These findings suggest that the delirium severity score provides an approach for measuring delirium severity that is associated with adverse clinical outcomes in a direct exposure-response association and that the delirium severity score may help advance patient-centered care for delirium.

## Key Points

**Question:**

Are scores on a delirium severity measure that was developed using advanced psychometric approaches associated with in-hospital and posthospital clinical outcomes?

**Findings:**

In this cohort study of older patients (aged ≥70 years), patients in the highest delirium severity score short form group (scores 6-9) had longer mean length of stay (13.3 vs 6.9 days), greater in-hospital costs ($57 700 vs $34 200), higher 1-year costs ($168 700 vs $106 500), and increased mortality at 1 year (50% vs 17%) compared with patients in the lowest delirium severity score short form group (score 0).

**Meaning:**

These findings suggest that the delirium severity score provides an approach for measuring delirium severity that is associated with adverse clinical outcomes and that the delirium severity score may help advance patient-centered care for delirium.

## Teaser

This cohort study examines the internal consistency, reliability, and validity for clinical outcomes of a measure of delirium severity that was developed using advanced psychometric approaches.

## Introduction

Delirium, an acute change in cognition, is a common, morbid, and costly syndrome, affecting up to 64% of older medical patients and up to 50% of older surgical patients.^1^ Delirium is associated with longer hospital stays,^2^ greater iatrogenic complications,^3^ higher rates of institutionalization,^4^ increased risks of incident dementia,^5^ and increased in-hospital mortality (25%-33%).^6,7^ The estimated annual US health care costs attributable to delirium range up to $182 billion (2011 US dollars), and estimates of $146 358 per year per patient are attributable to delirium, with postsurgery health care costs increasing substantially and directly with the level of delirium severity (no or mild delirium, $83 534 per patient per year; moderate delirium, $99 756 per patient per year; and severe delirium, $140 008 per patient per year).^8,9^ Moreover, with its increased incidence during the COVID-19 pandemic,^10,11^ delirium has garnered widespread attention as a worldwide public health and patient safety priority.^12,13,14^

Although identification of delirium has advanced with more than 30 instruments available,^15^ the importance of measuring delirium severity has received comparatively scant attention. Clinically, the ability to rate delirium severity in older adults is essential to providing nuanced and appropriate patient-centered care. Such ratings allow clinicians to identify the most severe delirium cases, to monitor response to treatment over time, and to provide more graded assessments of recovery time and prognosis, given the poor outcomes associated with severe delirium. Many clinicians recognize that simply characterizing delirium as either present or absent is insufficient to allow refined clinical input to formulate and evaluate clinical interventions.^16^ Moreover, such measures are already in demand to better assess nursing burden, safety concerns, and staffing needs across settings. Delirium severity measures are also needed to inform research, as graded outcome measures are necessary for better design of clinical trials, pathophysiological-mechanistic investigations, and prognostic studies. The Confusion Assessment Method–Severity (CAM-S) score,^17^ developed by our group in 2014, provided a severity measure that is associated with clinical outcomes and that has gained substantial use. However, the CAM-S does not include a cognitive assessment, patient self-report, or psychometrically derived items.

Our goal was to develop a new delirium severity measure, the DEL-S delirium-severity score, following state-of-the-art psychometric approaches, analogous to those used by the Patient-Reported Outcomes Measurement Information System initiative.^18^ Accordingly, we pursued the following multistep process: (1) systematic review of the medical literature on delirium severity and evaluation of the quality of existing tools^19^; (2) psychometric synthesis and harmonization of the 3 most commonly used delirium severity instruments using advanced psychometric methods to generate an item bank^20^ of delirium severity measures; (3) in-depth qualitative interviews with patients, caregivers, and nurses to ensure comprehensive inclusion of key domains of delirium severity^21^; (4) a modified Delphi process involving an interdisciplinary panel of delirium experts to define domains and subdomains of delirium severity that do not overweight hyperactive symptoms^22^; and (5) a prospective study to evaluate new delirium severity items using advanced measurement methods including item response theory.^23^ The resulting instrument includes cognitive test items, patient self-report, and observer-rated items. Here we present our new delirium severity measure, the delirium severity score, to describe its distribution and internal reliability and to evaluate its association with clinically relevant outcomes at hospital discharge and after hospitalization, including length of stay (LOS), hospital costs, health care costs, rehospitalization, and cumulative mortality at 30 days, 90 days, and 1 year.

## Methods

### Study Sample

Data were obtained from the Better Assessment of Illness study,^24^ which enrolled a prospective cohort of older adults hospitalized at the Beth Israel Deaconess Medical Center academic medical center in Boston, Massachusetts, from October 20, 2015, to March 15, 2017. The Better Assessment of Illness study^24^ was designed to develop and test new delirium severity measures, compare these measures with existing measures, and investigate their associations with clinical outcomes. The sample size was based on a priori calculations accounting for expected attrition. In brief, English-speaking participants aged 70 years and older who were admitted or transferred to the medical or surgical services as either emergency or elective admissions and lived within 40 miles of Beth Israel Deaconess Medical Center were enrolled. Exclusion criteria included the inability to participate in cognitive assessment because of legal blindness or severe deafness, history of heavy alcohol use or alcohol withdrawal within the last 6 months, diagnosis of schizophrenia or active psychosis, plans for immediate discharge, or admission for a terminal condition. Although the study was intended to be as inclusive as possible, patients with planned discharges within 48 hours were not eligible given the limited opportunity for assessment and low risk for delirium. Written informed consent was obtained from participants (whenever possible) or a health care proxy. Study procedures were approved by the institutional review boards of Beth Israel Deaconess Medical Center and Hebrew SeniorLife, the study coordinating center. This cohort study followed the Strengthening the Reporting of Observational Studies in Epidemiology (STROBE) reporting guideline for observational studies.

### Study Procedures

Interviewers conducted initial evaluations within 48 hours of hospital admission, followed by daily assessments throughout the hospitalization. The initial assessment collected information on demographics, delirium status, cognition, and other variables. Subsequently, delirium and delirium severity were assessed during daily in-person interviews. We conducted daily interviews for each patient on the first 4 days of their hospital stay. After day 4, interviews were conducted every other day until discharge, with the following exception: if delirium was present on any of the first 4 days of hospitalization, daily interviews were continued until discharge. There was no limit to the maximum number of assessments per patient. Research associates received extensive training and underwent semiannual standardization on key study measures.

### Delirium and Delirium Severity

Delirium was determined with cognitive testing and the Confusion Assessment Method (CAM)^25^ supplemented by a validated medical record review method.^26^ Patients were considered delirious if delirium was present either by the CAM diagnostic algorithm^25^ or medical record review by an experienced research physician. The combination of CAM and medical record review method maximizes sensitivity in detecting delirium.^26^

Delirium severity was quantified using the delirium severity score short-form (SF)^27^ and long-form (LF) score.^23,28^ The delirium severity score was developed using advanced measurement methods and used a panel of delirium experts tasked with identifying domains and indicators of delirium severity that provided broad domain coverage across delirium symptoms, yielded high construct and content validity, enabled quick administration, and would be easy to use by trained raters.^22,29^ The delirium severity score SF score is based on 6 observer-rated items (range, 0-13 points, with higher scores denoting worse delirium), and the delirium severity score LF is based on 17 observer-rated items (range, 0-21 points, with higher scores denoting worse delirium). See Vasunilashorn et al^22^ for specific items. We considered the peak delirium severity score, which was defined as the highest delirium severity score on any hospital day. On the basis of prior work comparing peak scores with 8 other methods for measuring the severity of an episode of delirium, we found that a measure capturing peak scores of delirium severity emerged as a preferred measure.^30^

### Outcomes

#### Hospital Outcomes

We considered 2 hospital outcomes: LOS and hospital costs. LOS (days) was determined by a standardized medical record review conducted after hospital discharge.^22^ Hospital costs were computed from Medicare Part A and B administrative claims, which include Medicare Provider Analysis and Review (MEDPAR), Home Health Agency, and outpatient files from 2015 to 2019. MEDPAR files include inpatient hospital and postacute skilled rehabilitation stays covered by Medicare. Home Health Agency files include services covered by Medicare Home Health Agencies. Outpatient files include fee-for-service claims billed by institutional outpatient health care workers. Total hospital costs incurred during the hospital stay were determined from MEDPAR. Costs were estimated using Medicare reimbursement amounts, rather than billing charges, because reimbursed amounts indicate the actual payments received by clinicians and hospitals and better represent transaction costs.^31^ We report costs in 2019 US dollars, adjusting for inflation using the Consumer Price Index medical care component.^32^

#### Posthospital Outcomes

We considered 3 posthospital outcomes for 30 days and 1 year after hospitalization: death, posthospital costs not including the index surgery costs, and rehospitalization (additionally considering 90-day rehospitalization), which is considered a hospital quality measure by the Centers for Medicare & Medicaid Services.^33^ Information on death was ascertained by caregiver report, medical record review, obituary review, and the National Death Index. Cumulative health care costs within 30 days and 365 days were calculated as a total of all post–index hospitalization health care cost categories (eg, postacute rehabilitation facility, home health care, and outpatient). Rehospitalization within 90 days was determined by patient and caregiver report and confirmed by Medicare data. See eFigure 1 in Supplement 1 for a breakdown of the available study data.

### Descriptive and Control Variables

Age, sex, race and ethnicity (American Indian or Alaska Native, Asian, Black or African American, Hispanic ethnicity, White, and more than 1 race), and admission service were determined from the patient’s medical records. Race and ethnicity were assessed in this study to consider potential differences in delirium severity score by hospital and posthospital outcomes. Other study variables from patient interviews included education, marital status, living situation, activities of daily living, and the Montreal Cognitive Assessment.^34^ Dementia and mild cognitive impairment (MCI) were defined according to a systematic medical record review process described elsewhere.^24^ In brief, relevant medications, neuroimaging studies, practitioner notes (inpatient and outpatient) documenting a diagnostic evaluation for dementia, memory loss, or formal neuropsychological testing were extracted. Medical record evidence of MCI or dementia required documentation of cognitive impairment from at least 2 separate sources, and uncertain cases were adjudicated by clinical experts.

### Internal Consistency Reliability and Interrater Reliability

To assess internal consistency, we examined the McDonald ω coefficient^35,36^ with 95% CIs generated with bootstrap resampling. To assess interrater reliability, a total of 42 paired delirium severity score ratings were conducted, with 2 observers rating each patient simultaneously in a blinded manner. Interrater agreement was assessed with a weighted κ statistic. We used quadratic weights so that the κ statistics can be interpreted on the same scale as the intraclass correlation coefficient.^37^ The 95% CIs were obtained using bootstrap methods. See the eAppendix in Supplement 1 for additional details.

### Statistical Analysis

To enhance interpretability for analyses related to clinical outcomes, we divided the delirium severity scores into 5 categories. The lowest level included no delirium symptoms (delirium severity score of 0), and the second lowest level represented subsyndromal delirium (delirium severity score of 1, the minimum among CAM-positive patients). The remaining 3 categories were defined empirically according to the score distributions in patients with CAM-positive delirium: category 3 included delirium severity scores in the lower third among the CAM-positive patients with delirium, and categories 4 and 5 included delirium severity scores in the middle third and top third, respectively, among the CAM-positive patients with delirium. The delirium severity score quintiles were developed by first using the cut points for delirium severity developed from a prior study^38^ and then subdividing the large no-delirium group to yield the 5-category distribution. See the eAppendix in Supplement 1 for distribution of categories by delirium status.

We display the means of the outcome variables across delirium severity categories characterized with the delirium severity score (SF and LF). To characterize trends and differences, we used Gaussian generalized linear models with a log link to characterize the association between delirium severity score (SF and LF) and hospital costs (in-hospital and 30 days and 1 year after hospitalization). *P* values for the linear trend in the association between the delirium severity scores and each outcome were obtained by running the same analytical models with the variable for the delirium severity score included as a continuous measure (as opposed to a categorical measure). For LOS, rehospitalization (90 days and 1 year after hospitalization), and death (30 days and 1 year after hospitalization), we used Poisson regression with robust variance estimation to characterize the risk of any rehospitalization or risk of death. We used inverse propensity score weights to account for missing data.^39^ This approach to missingness uses the complete case data and uses a propensity score weighting approach to address selection effects to the complete case sample (see the eAppendix in Supplement 1 for details on missing observations for our outcome variables).

We used models to characterize the linear association between outcomes and delirium severity by including a rank-based normalization transformation of the delirium severity measure as a linear indicator of the outcome.^40^ We report fold differences (and 95% CIs) between delirium severity score categories for our continuous outcomes (ie, LOS and hospital costs) and report relative risks (RR) and 95% CIs for our dichotomous outcomes (ie, death and rehospitalization). The interpretations of fold differences and RRs, as well as their corresponding 95% CIs, are similar, with the fold differences indicating how much the mean of a given delirium severity score category differs from another category (see the eAppendix in Supplement 1 for details on fold differences).

We compared the RR implied by the models in terms of the estimated level of the outcome in the lowest severity category and higher severity categories. All models were adjusted for age, sex, race or ethnicity, dementia, or MCI at study enrollment. All analyses were conducted with Stata statistical software version 16.1 (StataCorp). The 95% CIs for fold differences were estimated with the Stata nlcom command, and the Stata margins command was used to obtain estimates of adjusted means and SDs. A 2-tailed α level of .05 was used as a guide to statistical significance. Data analysis was performed from June 2020 to August 2021.

## Results

Table 1 shows the characteristics of the study sample of 352 participants. Patients had a median (IQR) age of 79.7 (74.6-85.5) years, with a median (IQR) of 14 (7-20) years of education, and a median (IQR) baseline Montreal Cognitive Assessment score of 18.5 (15.0-21.0). Patients were mostly female (204 women [58.0%]) and White (300 patients [85.2%]); 139 patients (39.5%) were currently married or living with a partner, 69 (19.6%) had delirium, and 85 (24.1%) had dementia or MCI at enrollment.

**Table 1.** Sample Characteristics

Characteristic	Patients, No. (%) (N = 352)
Age, median (IQR), y	79.7 (74.6-85.5)
Sex	
Female	204 (58.0)
Male	148 (42.0)
Race and ethnicity	
White	300 (85.2)
Other racial and ethnic groups^a^	52 (14.8)
Duration of education, median (IQR), y	14 (7-20)
Currently married or living with a partner	139 (39.5)
Living alone	135 (39.4)
Living in nursing home	13 (3.7)
Surgical patient	102 (29.0)
ADL impairment	
Any ADL impairment	272 (77.3)
ADL score, median (IQR)^b^	3 (1-5)
Delirium ever during hospitalization	69 (19.6)
Baseline Montreal Cognitive Assessment score, median (IQR)	18.5 (15.0-21.0)
Dementia or mild cognitive impairment at time of enrollment	85 (24.1)

Abbreviation: ADL, activities of daily living.

^a^ Other refers to American Indian or Alaska Native, Asian, Black or African American, Hispanic ethnicity, and more than 1 race.

^b^ Score range is 0 to 14, with higher score indicating worse impairment.

Table 2 displays the means or proportions for the delirium severity score SF and LF groups among the 1190 total delirium severity score observations in the 352 patients. For the delirium severity score SF, 452 patients (38.0%) had a delirium severity score score of 0, 344 (28.9%) had a delirium severity score of 1, 244 (20.5%) had a delirium severity score of 2 to 3, 90 (7.6%) had a delirium severity score of 4 to 5, and 60 (5.0%) had a delirium severity score of 6 to 9 points. For the delirium severity score LF, 417 patients (35.1%) had a delirium severity score of 0, 308 (26.0%) had a delirium severity score of 1, 311 (26.2%) had a delirium severity score of 2 to 4, 83 (7.0%) had a delirium severity score of 5 to 6, and 68 (5.7%) had a delirium severity score of 7 to 14 points. All patients who experienced delirium by CAM had delirium severity score (SF and LF) scores of 2 points or higher. eFigure 2 in Supplement 1 illustrates the distributions of delirium severity score SF and LF scores.

**Table 2.** Categorization of DEL-S Short Form and Long Form Among the Total Number of Observations From 352 Patients Stratified by Delirium Status

DEL-S form and scores	Observations, No. (%)^a^		
	Total (N = 1190)	Delirium (n = 167)	No delirium (n = 1020)
DEL-S short form			
0	452 (38.0)	0	449 (44.0)
1	344 (28.9)	0	344 (33.7)
2-3	244 (20.5)	45 (27.0)	199 (19.5)
4-5	90 (7.6)	65 (38.9)	25 (2.5)
6-9	60 (5.0)	57 (34.1)	3 (0.3)
DEL-S long form			
0	417 (35.1)	0	417 (40.9)
1	308 (26.0)	0	308 (30.2)
2-4	311 (26.2)	44 (26.4)	267 (26.2)
5-6	83 (7.0)	57 (34.1)	26 (2.5)
7-14	68 (5.7)	66 (39.5)	2 (0.2)

Abbreviation: DEL-S, delirium-severity score.

^a^ Differences in the sample size between the total column and delirium status columns reflect missing Confusion Assessment Method (CAM)–positive or CAM-negative designations because of missing data. There were 3 participants who did not have a DEL-S long form or CAM algorithm completed.

Delirium severity score SF and LF scores demonstrated high to very high internal consistency reliability (McDonald ω coefficient, 0.89 [95% CI, 0.85-0.92] for SF and 0.94 [95% CI, 0.92-0.95] for LF), supporting individual level inferences.^33^ The interrater agreement for the delirium severity score categories demonstrated high concordance (κ = 0.72 [95% CI, 0.52-0.87] for SF and κ = 0.74 [95% CI, 0.55-0.86] for LF) and high absolute agreement (94% for SF and 95% for LF). See the eAppendix in Supplement 1 for detailed results.

Table 3 shows the associations between delirium severity scores and hospital outcomes, with significant associations found between increasing scores and worse outcomes. Each increase in delirium severity score category for both SF and LF was associated with an increase in mean LOS, adjusting for age, sex, race and ethnicity, and dementia or MCI at study enrollment. Patients in the highest delirium severity score SF category (score 6-9 points) had an mean LOS of 13.3 days (95% CI, 9.8-16.7 days), nearly double (13.3 / 6.9 = 1.9 times; 95% CI, 1.3-2.5) the mean LOS of patients with a delirium severity score SF of 0 points, at 6.9 days (95% CI, 5.8-7.9 days) (*P* for trend < .001). Similarly, patients in the highest delirium severity score LF category (score 7-14 points) had a mean LOS of 12.5 days (95% CI, 9.6-15.3 days) compared with 6.7 days (95% CI, 5.6-7.9 days) for those with delirium severity scores of 0 points (*P* < .001) (fold difference, 12.5 / 6.7 = 1.9; 95% CI, 1.3-2.4). Adjusted mean hospital costs yielded similar results; patients in the highest delirium severity score category had significantly greater hospital costs than patients in the lowest category (delirium severity score of 0): $57 700 (95% CI, $41 800-$73 700) for delirium severity SF score 6 to 9 points vs $34 200 (95% CI, $22 900-$45 500) for delirium severity SF score 0 points (fold difference, 2.0; 95% CI, 1.1-3.0), and $51 700 (95% CI, $36 500-$66 900) for delirium severity LF score 7 to 14 points vs $36 000 (95% CI, $23 600-$48 400) for delirium severity LF score 0 points (fold difference, 1.7; 95% CI, 0.9-2.6). See the eAppendix in Supplement 1 for additional analytical results.

**Table 3.** Association of DEL-S Score With Hospital Outcomes^a^

DEL-S score	Patients, No.	Adjusted mean (95% CI)^b^	
		Length of stay, d (n = 352)	Hospital costs, $US in thousands (n = 299)
DEL-S short form			
0	64	6.9 (5.8-7.9)	34.2 (22.9-45.5)
1	92	7.5 (6.6-8.4)	38.7 (29.5-47.9)
2-3	86	9.5 (8.0-11.1)	41.2 (31.8-50.6)
4-5	37	9.7 (8.1-11.3)	36.4 (22.5-50.4)
6-9	29	13.3 (9.8-16.7)	57.7 (41.8-73.7)
* P* value for trend^c^	NA	<.001	.004
DEL-S long form			
0	55	6.7 (5.6-7.9)	36.0 (23.6-48.4)
1	88	7.4 (6.6-8.3)	38.7 (29.3-48.1)
2-4	94	9.3 (8.0-10.7)	40.8 (31.7-49.8)
5-6	38	9.8 (7.7-11.9)	37.4 (23.1-51.6)
7-14	33	12.5 (9.6-15.3)	51.7 (36.5-66.9)
* P* value for trend^c^	NA	<.001	.01

Abbreviations: DEL-S, delirium-severity score; NA, not applicable.

^a^ Maximum (peak) Confusion Assessment Method–Severity score during each patient’s hospitalization was used in all analyses.

^b^ Models were adjusted for age, sex, race and ethnicity, dementia, or mild cognitive impairment at time of enrollment.

^c^ The *P* value for trend is a linear trend statistic derived from general linear models with a log-link function for the association of the DEL-S with each outcome.

The associations between delirium severity score and posthospital outcomes at 30 and 90 days are presented in Table 4, demonstrating significant associations between increasing scores and worse posthospital outcomes. The highest proportion of deaths 30 days after hospital discharge was observed for patients in the highest delirium severity score category (SF and LF). Four of 34 patients (11.8%) with delirium severity SF scores of 6 to 9 points died compared with 5 of 75 patients (6.7%) with a delirium severity SF score of 0 points (RR, 1.6; 95% CI, 0.3-11.6); 6 of 39 patients (15.4%) with delirium severity LF scores of 7 to 14 points died compared with 4 of 64 patients (6.3%) with a delirium severity LF score of 0 points (RR, 2.3, 95% CI, 0.5-13.1). Similarly, the highest adjusted (for age, sex, race and ethnicity, and dementia or MCI at study enrollment) mean hospital costs between hospital discharge to 30 days were observed among patients in the highest delirium severity score category. Mean costs were $14 200 (95% CI, $8200-$20 200) for patients with delirium severity SF scores of 6 to 9 points compared with $9300 (95% CI, $5200-$13 500) for patients with DELS-S SF score 0 points (fold difference, 1.8; 95% CI, 0.6-2.9) and $17 000 (95% CI, $11 200-$22 700) for patients with delirium severity LF scores of 7 to 14 points compared with $10 000 (95% CI, $5500-$14 500) for patients with delirium severity LF score 0 points (fold difference, 2.0; 95% CI, 0.8-3.3). See the eAppendix in Supplement 1 for additional analytical results.

**Table 4.** Association of DEL-S Score With Posthospital Outcomes at 30 and 90 Days^a^

DEL-S score	Death within 30 d, patients, No./total No. (%) (n = 352)^b^	Adjusted cumulative health care costs to 30 d, mean (95% CI), $US in thousands (n = 308)^b^	Rehospitalization within 90 d, patients, No./total No. (%) (n = 352)^b^
DEL-S short form			
0	5/75 (6.7)	9.3 (5.2-13.5)	22/63 (35.1)
1	4/108 (3.7)	11.6 (8.0-15.1)	30/93 (32.2)
2-3	4/91 (4.4)	11.2 (7.6-14.7)	37/88 (42.0)
4-5	3/44 (6.8)	17.7 (12.4-23.1)	18/37 (48.6)
6-9	4/34 (11.8)	14.2 (8.2-20.2)	12/30 (39.7)
* P* value for trend^c^	.42	.03	.57
DEL-S long form			
0	4/64 (6.3)	10.0 (5.5-14.5)	20/53 (37.9)
1	5/104 (4.8)	12.3 (8.7-15.9)	27/90 (30.0)
2-4	3/101 (3.0)	10.4 (6.9-13.9)	41/96 (42.7)
5-6	2/44 (4.5)	13.9 (8.5-19.2)	15/39 (38.3)
7-14	6/39 (15.4)	17.0 (11.2-22.7)	16/33 (48.2)
* P* value for trend^c^	.39	.06	.58

Abbreviation: DEL-S, delirium-severity score.

^a^ Maximum (peak) Confusion Assessment Method–Severity score during each patient’s hospitalization was used in all analyses.

^b^ Models were adjusted for age, sex, race and ethnicity, dementia, or mild cognitive impairment at time of enrollment.

^c^ The *P* value for trend is a linear trend statistic derived from general linear models with a log-link function for the association of the DEL-S with each outcome.

The association between delirium severity scores and longer term outcomes (1 year) is reported in Table 5, analyses reflect adjustment for age, sex, race or ethnicity, and dementia or MCI at study enrollment. Significant trends were demonstrated between increasing scores and adverse outcomes. No patients were missing outcome data on mortality. Patients in the highest delirium severity score category demonstrated the highest mortality rate, greatest mean hospital costs, and highest proportion of rehospitalization within 1 year. Seventeen of 34 patients (50.0%) with a delirium severity score of 6 to 9 points died within 1 year compared with 13 of 75 patients (17.3%) with SF scores of 0 points, a greater than 3-fold difference (RR, 3.7; 95% CI, 0.06-7.26). These proportions were similar for the delirium severity score LF: 18 of 39 patients (46.2%) with scores of 7 to 14 compared with 12 of 64 patients (18.8%) with a score of 0 died within 1 year (RR, 2.7; 95% CI, 0.1-5.4). Patients in the highest delirium severity score category had the highest rate of 1-year rehospitalization: 23 of 30 patients (76.7%) with delirium severity SF score 6 to 9 points vs 38 of 63 patients (60.6%) with delirium severity SF score 0 points (fold difference, 1.2; 95% CI, 0.6-1.9) and 27 of 33 patients (81.9%) with delirium severity LF score 7 to 14 points vs 33 of 53 patients (62.6%) with delirium severity LF score 0 points (fold difference, 1.3; 95% CI, 0.6-2.0). The highest mean cumulative health care costs were observed for patients in the highest delirium severity score categories: $168 700 (95% CI, $124 900-$212 400) for delirium severity SF scores 6 to 9 points vs $106 500 (95% CI, $75 600-$137 400) for SF score 0 points (fold difference, 1.8; 95% CI, 1.1-2.5) and $158 400 (95% CI, $117 000-$200 000) for delirium severity LF scores 7 to 14 points vs $106 100 (95% CI, $72 800-$139 400) for LF score 0 points (fold difference, 1.7; 95% CI, 0.96-2.4). For additional analytical results, refer to the eAppendix in Supplement 1.

**Table 5.** Association of DEL-S Score With Posthospital Outcomes at 1 Year^a^

DEL-S score	Patients, No./total No. (%)		Adjusted cumulative health care costs to 1 y, mean (95% CI), $US in thousands (n = 311)^b^
	Death within 1 y (n = 352)^b^	Rehospitalization within 1 y (n = 352)^b^	
DEL-S short form			
0	13/75 (17.3)	38/63 (60.6)	106.5 (75.6-137.4)
1	23/108 (21.3)	53/93 (57.0)	104.7 (78.8-130.5)
2-3	27/91 (29.7)	55/88 (62.4)	118.6 (92.5-144.7)
4-5	12/44 (27.3)	28/37 (75.7)	122.9 (83.2-162.4)
6-9	17/34 (50.0)	23/30 (76.7)	168.7 (124.9-212.4)
* P* value for trend^c^	.02	.21	.03
DEL-S long form			
0	12/64 (18.8)	33/53 (62.6)	106.1 (72.8-139.4)
1	22/104 (21.2)	51/90 (56.7)	105.0 (78.4-131.6)
2-4	28/101 (27.7)	59/96 (61.3)	123.3 (98.0-148.7)
5-6	12/44 (27.3)	27/39 (68.7)	111.3 (71.1-151.4)
7-14	18/39 (46.2)	27/33 (81.9)	158.4 (117.0-200.0)
* P* value for trend^c^	.03	.19	.01

Abbreviation: DEL-S, delirium-severity score.

^a^ Maximum (peak) Confusion Assessment Method–Severity score during each patient’s hospitalization was used in all analyses.

^b^ Models were adjusted for age, sex, race and ethnicity, dementia, or mild cognitive impairment at time of enrollment.

^c^ The *P* value for trend is a linear trend statistic derived from general linear models with a log-link function for the association of the DEL-S with each outcome.

## Discussion

This cohort study provides evidence supporting the usefulness and validity of a novel delirium severity measure, the delirium severity score. The measure demonstrates strong psychometric properties, including internal reliability and interrater reliability, along with associations with clinical outcomes related to delirium severity. Importantly, the delirium severity score was significantly associated with hospital outcomes (LOS and hospital costs) and posthospital outcomes (mortality at 1 year and health care costs at 30 days and 1 year) in a direct exposure-response association.

Although this study builds on prior delirium severity instruments, including the CAM-S,^17^ Delirium Rating Scale-R98,^41^ and Memorial Delirium Assessment Scale,^42^ the delirium severity score enhances the prior work by using state-of-the-art measurement approaches, combining expert input and item response theory–based modeling to formulate final items. The items adequately represent hypoactive delirium, which is often overlooked. The delirium severity score provides the cognitive assessment and self-report items, as well as finely graded observer ratings, advancing the scope of the CAM-S. Other strengths include the rigorous and prospective development of the delirium severity score through a 5-year multistep process, and the clinical outcomes derived from long-term follow-up of the cohort provide evidence for the validity of the delirium severity score.

The delirium severity score has both a SF (6 item rating) and a LF (17 item rating). With its brevity, the delirium severity score SF will be preferred for clinical use, such as for assessing patients over time or for assigning nurse staffing according to patient acuity. With superior range and distinction between categories, the delirium severity score LF will be valuable for clinical research including treatment trials or pathophysiologic studies, where the ability to measure subtle changes over time or nuanced relationships with biomarkers may be critical. Both instruments are freely available online.^27,28^

### Limitations

Several important caveats should be noted. First, the delirium severity score items were selected from a larger battery and, thus, have not been validated in the exact format presented. Thus, the final instrument may need to be adapted for optimal use. Second, the delirium severity score was derived in a single-site study, and, thus, further validation studies in other study populations and settings will be key to ensure generalizability. Third, given the small number of outcomes in some strata, our study may have had limited power to detect significant differences. Fourth, we were parsimonious in the control variables in our models and did not include many that were considered as potential mediators between delirium and clinical outcomes (eg, comorbid illnesses and polypharmacy). Fifth, the delirium severity score has not been validated for identification of delirium, and simply selecting a cut point for identification is not recommended at this time. Thus, an additional instrument is required to initially identify the presence of delirium.

## Conclusions

The delirium severity score provides an important and novel tool for measuring delirium severity, which is associated with multiple adverse clinical outcomes and health care costs. As such, the delirium severity score will help to optimize delirium management clinically, with potential financial and quality of care implications. Moreover, it may provide a useful outcome measure for clinical trials or biomarker studies in delirium. In an independent prospective study (under way), we will administer the delirium severity score instrument and prospectively compare the delirium severity score with reference standard ratings and other delirium severity measures. Future work will be essential to validate its use for identification of delirium and to validate its usefulness in other samples and settings.

## Notes

Supplement 1. **eAppendix. **Supplemental Methods and Results **eFigure 1.** Breakdown of the Available Study Data **eFigure 2.** Histograms of DEL-S
eReferences

Supplement 2.
BASIL Study Team
