# Supplementary material for: Psychometric Properties of a Delirium Severity Score for Older Adults and Association With Hospital and Posthospital Outcomes
Source: JAMA Netw Open. 2022 Mar 31;5(3):e226129. doi: 10.1001/jamanetworkopen.2022.6129 (PMC8972033; doi:10.1001/jamanetworkopen.2022.6129)
Supplement: Supplement 1. — eAppendix. Supplemental Methods and Results eFigure 1. Breakdown of the Available Study Data eFigure 2. Histograms of DEL-S eReferences [file jamanetwopen-e226129-s001.pdf]

Supplemental Online Content

Vasunilashorn SM, Fong TG, Helfand BKI, et al; BASIL Study Team. Psychometric properties of a delirium severity score for older adults and association with hospital and posthospital outcomes. *JAMA Netw Open*. 2022;5(3):e226129. doi:10.1001/jamanetworkopen.2022.6129

eAppendix. Supplemental Methods and Results
eFigure 1. Breakdown of the Available Study Data
eFigure 2. Histograms of DEL-S
eReferences

This supplemental material has been provided by the authors to give readers additional information about their work.

© 2022 Vasunilashorn SM et al. *JAMA Network Open.*

eAppendix. Supplemental Methods and Results

Analysis notes

Fold difference

The manuscript reports “fold-difference” effect size statistics (we don’t use the more commonly encountered language “fold-change” because we are describing group differences, not changes). The fold-difference is computed after regression models and reflect adjustment for covariates and missing data handling. The fold-difference is computed, generally, as the expected value among persons in the highest severity category divided by the expected value among persons in the lowest severity category, with covariates held at their overall sample means. This is, in general, (b0 + b4)/b0 where b0 is the regression model constant term (the expected value in the reference group, as all covariates are mean centered) and b4 is the regression parameter associated with being in the highest category of severity. An alternative form is exp(b0 + b4)/exp(b0) for outcomes that are modeled on a log scale (including Poisson [length of stay] and log normal [costs] out- comes). interval estimates are obtained using the Delta method.

Here we explain how this works. First, consider the length of stay variable according to levels of severity on the long form of the DELS:

```
. table dels17cat_peak , c(n vdlos mean vdlos) f(%5.3f)

------------------------------------------
DELS Long Form   |
category         |
(peak)           |    N(vdlos)  mean(vdlos)
-----------------+------------------------
      DELS17: 0  |          64        7.125
      DELS17: 1  |         104        7.471
  DELS17: 2 - 4  |         101        9.139
  DELS17: 5 - 6  |          44        9.523
 DELS17: 7 - 14  |          39       12.103
------------------------------------------
```

The mean length of stay in the high severity group (12.103) is 1.699 times the mean length of stay in the low severity group (7.125). If we look at a regression model comparing length of stay controlling for our select covariates:

```
. poisson vdlos i.dels17cat_peak cvdagebaseline cvdfemale cvdnonwhite
cvdcogimpair , vce(robust)Iteration 0:     log pseudolikelihood =  -1246.262
Iteration 1:   log pseudolikelihood = -1246.2617
Iteration 2:   log pseudolikelihood = -1246.2617

Poisson regression                              Number of obs     =        352
                                                Wald chi2(8)      =      34.38
                                                Prob > chi2       =     0.0000
Log pseudolikelihood = -1246.2617               Pseudo R2         =     0.0524

--------------------------------------------------------------------------------
               |               Robust
         vdlos |      Coef.   Std. Err.      z    P>|z|     [95% Conf. Interval]
---------------+----------------------------------------------------------------
dels17cat_peak |
     DELS17: 1 |   .1028897    .107599     0.96   0.339    -.1080005    .3137798
 DELS17: 2 - 4 |   .3277457     .11289     2.90   0.004     .1064853    .5490061
 DELS17: 5 - 6 |   .3762319     .14685     2.56   0.010     .0884112    .6640525
DELS17: 7 - 14 |   .6201616   .1498899     4.14   0.000     .3263827    .9139404
               |
cvdagebaseline |  -.0049817   .0061442    -0.81   0.417    -.0170241    .0070606
     cvdfemale |   .0486041   .0788592     0.62   0.538    -.1059572    .2031653
```

© 2022 Vasunilashorn SM et al. *JAMA Network Open.*

cvdnonwhite	-.3505541	.1015694	-3.45	0.001	-.5496264	-.1514817
cvdcogimpair	-.1212554	.1049626	-1.16	0.248	-.3269783	.0844675
_cons	1.895645	.0890676	21.28	0.000	1.721076	2.070214

© 2022 Vasunilashorn SM et al. *JAMA Network Open.*

The expected value for mean length of stay, holding constant (more exactly, holding to the overall sample means of the covariates [the "c" prefix on the covariates indicates they have been overall sample mean centered]) is

```
. * expected value for low severity group
. nlcom exp(_b[_cons])
      _nl_1:  exp(_b[_cons])
```

vdlos	Coef.	Std. Err.	z	P>|z|	[95% Conf.	Interval]
_nl_1	6.65684	.5929087	11.23	0.000	5.49476	7.81892

```
. * expected value for high severity group
.  nlcom  exp(_b[_cons]+_b[4.dels17cat_peak])
      _nl_1: exp(_b[_cons]+_b[4.dels17cat_peak])
```

vdlos	Coef.	Std. Err.	z	P>|z|	[95% Conf.	Interval]
_nl_1	12.37659	1.434737	8.63	0.000	9.564553	15.18862

and their ratio is:

```
.  nlcom  exp(_b[_cons]+_b[4.dels17cat_peak])/exp(_b[_cons])
      _nl_1:  exp(_b[_cons]+_b[4.dels17cat_peak])/exp(_b[_cons])
```

vdlos	Coef.	Std. Err.	z	P>|z|	[95% Conf.	Interval]
_nl_1	1.859228	.2786796	6.67	0.000	1.313026	2.40543

Missing data: frequency and handling

In all cases, missing data in outcome variables was addressed with inverse propensity weighting. This means the probability of being missing on the outcome was modeled (using logistic regression) and the same predictors (including DEL-S) and the baseline value of the outcome used as predictors according to the main analysis model. The model was used to generate predicted prob- abilities of missing on the outcome given baseline and covariates, which were converted to inverse propensity weights using actual missingness. The inverse propensity weights were scaled to the total sample size among those non-missing on the outcome, and used to weight the sample before assessing the strength of association with the DEL-S. Please see Seaman & White (2013) for a discussion of the approach.

Missing observations for outcome variables

Outcome	N missing	%
Length of stay	0	0
Total health care cost, index hospitalization, in 2019 dollars	53	15
Total costs at 1 year	41	12
Total cost, discharge to 30 days	44	12
Any rehospitalization within 90 days	41	12
Any rehospitalization within 365 days	41	12
Dead within 30 days	0	0
Dead within 365 days	0	0

© 2022 Vasunilashorn SM et al. *JAMA Network Open.*

DEL-S component items

DELS Long Form

The DELS Long Form is constructed from 17 items. They are:

camlf2a	CAM: Inattention
camlf3a	CAM: Disorganized thinking
camlf4a	CAM: Altered level of consciousness
camlf5a	CAM: Disorientation
camlf8a	CAM: Psychomotor agitation
camlf8d	CAM: Psychomotor retardation
dsi3	DSI: Saw things not there
dsi4	DSI: Heard things not there anxty5 Patient act frightened [NPI-C]
dsi2	DSI: Felt confused
dsi5	DSI: Misinterpret a sound
dsi6	DSI: Things look distorted dsi7 DSI: Things not moving
dsi8	DSI: Think people try to harm seva2 Easily distracted by stimuli [DOS]
sevb3	Inappropriately startled by stimuli [DSI]
sevg2	Patient believe what interviewer know are not true

DELS Short Form

The DELS Short Form is constructed from 8 items. They are:

camlf2a	CAM: Inattention
camlf3a	CAM: Disorganized thinking
camlf4a	CAM: Altered level of consciousness
camlf5a	CAM: Disorientation
camlf8a	CAM: Psychomotor agitation
camlf8d	CAM: Psychomotor retardation
dsi3	DSI: Saw things not there
dsi4	DSI: Heard things not there

© 2022 Vasunilashorn SM et al. *JAMA Network Open.*

DEL-S Categories

This section describes categorization of delirium severity using the DEL-S using the distribution of daily DEL-S scores. Therefore, this categorization is indifferent to peak and sums. The point is to arrive at a categorization that can be interpreted at the bedside, not after discharge.

Participants are grouped into five levels of severity according to both instruments. The five levels of severity are defined as:

DELS cat level	DELS6 sums	DELS17 sums	Threshold definition
0	0	0	No DELS symptoms (sum score is 0)
1	1	1	DELS sum is not 0 but less than minimum among CAM delirium positive
2	2 - 3	2 - 4	DELS sum is in the lower third among CAM delirium positive
3	4 - 5	5 - 6	DELS sum is in the middle third among CAM delirium positive
4	6 - 9	7 - 14	DELS score is in the top third among CAM delirium positive

Table
DELS categories by VDCAMDELIRIUM status

	Total		No		Yes	
**Characteristic**	**Mean or *n***	**(SD or %)**	**Mean or *n***	**(SD or %)**	**Mean or *n***	**(SD or %)**
Number of observations [N (%)]	1190	(100)	1020	(100)	167	(100)
DELS Long Form category [n (%)]						
DELS17: 0	417	(35·1)	417	(40·9)	0	(0·0)
DELS17: 1	308	(25·9)	308	(30·2)	0	(0·0)
DELS17: 2 - 4	311	(26·2)	267	(26·2)	44	(26·3)
DELS17: 5 - 6	83	(7·0)	26	(2·5)	57	(34·1)
DELS17: 7 - 14	68	(5·7)	2	(0·2)	66	(39·5)
DELS Short Form category [n (%)]						
DELS6: 0	452	(38·0)	449	(44·0)	0	(0·0)
DELS6: 1	344	(28·9)	344	(33·7)	0	(0·0)
DELS6: 2 - 3	244	(20·5)	199	(19·5)	45	(26·9)
DELS6: 4 - 5	90	(7·6)	25	(2·5)	65	(38·9)
DELS6: 6 - 9	60	(5·0)	3	(0·3)	57	(34·1)

© 2022 Vasunilashorn SM et al. *JAMA Network Open.*

Results

Sample descriptive statistics

Characteristic	Mean (SD) or n (%)		Observed range
Total [n (%)]	352	(100)	
Age at This Visit [Median (P'tile 25-75)]	79.7	[74.6-85.5]	[70.0-98.1]
Sex [n (%)]			
Men	148	(42.0)	
Women	204	(58.0)	
Race [n (%)]			
White	300	(85.2)	
All other race and ethnicity groups	52	(14.8)	
Currently Married Living with Partner [n (%)]			
No	211	(60.3)	
Yes	139	(39.7)	
Lives Alone [n (%)]			
No lives alone	215	(61.4)	
Yes	135	(38.6)	
Respondent s Education Years [Median (P'tile 25-75)]	14.0	[12.0-16.0]	[7.0-20.0]
Any ADL impairment [n (%)]			
None	70	(20.5)	
At least some ADL impairment	272	(79.5)	
ADL score [0-14] (higher worse) [Median (P'tile 25-75)]	3.0	[1.0-5.0]	[0.0-10.0]
CAM Delirium - Ever [n (%)]			
No	283	(80.4)	
Yes	69	(19.6)	
MOCA (without recall items prorated) [Median (P'tile 25-75)]	18.5	[15.0-21.0]	[0.0-25.0]
Dementia and MCI at time of enrollment [n (%)]			
No	267	(75.9)	
Yes	85	(24.1)	

© 2022 Vasunilashorn SM et al. *JAMA Network Open.*

Internal consistency reliability

In our previous paper describing the item selection for the items in the DELS, we reported the internal consistency reliability using McDonald's omega coefficient. In this manuscript, we report the internal consistency for the short form item set as well. Coefficient omega is estimated based on the magnitude of the factor loadings from a single domain confirmatory factor analysis model, using estimation procedures appropriate for the ordinal nature of the response data. Confidence intervals are obtained using the bootstrap.

The short form has an omega coefficient of 0.89, (and a 95% CI of 0.85, 0.92), which is also very high and near the minimum level recommended to support individual level inferences (.90, Nunally & Bernstein, 1994).

The long form has an omega coefficient of 0.94, and a 95% CI of 0.92-0.95.

© 2022 Vasunilashorn SM et al. *JAMA Network Open.*

Inter-rater agreement (kappa)

Long form

```
. tab dels171_cat dels172_cat
```

DELS-17 categories (rater 1)	DELS-17 categories (rater 2) 0	1	2/4	5/6	7/14	Total
0	2	1	0	0	0	3
1	1	3	3	0	0	7
2/4	0	0	3	2	2	7
5/6	0	0	0	0	2	2
7/14	0	0	0	1	1	2
Total	3	4	6	3	5	21

```
. kap dels171_cat
dels172_cat , wgt(kwr5)
Ratings weighted by:
   1.0000   0.9380   0.7500   0.4380   0.0000
 0.9380   1.0000  0.9380  0.7500  0.4380
 0.7500   0.9380  1.0000  0.9380  0.7500
 0.4380   0.7500  0.9380  1.0000  0.9380
 0.0000   0.4380  0.7500  0.9380  1.0000
```

Agreement	Expected Agreement	Kappa	Std. Err.	Z	Prob>Z
94.67%	79.19%	0.7437	0.2002	3.72	0.0001

Short form

```
. tab dels61_cat dels62_cat
```

DELS-6 categories (rater 1)	DELS-6 categories (rater 2) 0	1	2/3	4/5	6/9	Total
0	2	1	0	0	0	3
1	1	3	3	0	0	7
2/3	0	1	2	2	2	7
4/5	0	0	0	0	2	2
6/9	0	0	0	1	1	2
Total	3	5	5	3	5	21

```
. kap dels61_cat
dels62_cat , wgt(kwr5)
Ratings weighted by:
   1.0000   0.9380   0.7500   0.4380   0.0000
 0.9380   1.0000  0.9380  0.7500  0.4380
 0.7500   0.9380  1.0000  0.9380  0.7500
 0.4380   0.7500  0.9380  1.0000  0.9380
 0.0000   0.4380  0.7500  0.9380  1.0000
```

Agreement	Expected Agreement	Kappa	Std. Err.	Z	Prob>Z
94.37%	79.09%	0.7308	0.2024	3.61	0.0002

95% CI on kappa

Confidence intervals are obtained using bootstrap resampling. The 95% CI on weighted kappa forthe short form is: 0.56, 0.88

The 95% CI on weighted kappa for the long form is: 0.58, 0.89.

© 2022 Vasunilashorn SM et al. *JAMA Network Open.*

Length of stay - DELS Long Form

Differences across levels of delirium severity categories are tested with Poisson regression.

Fold difference: The expected length of stay was 1.859 times as long for those in the highest severity category relative to the lowest severity cate- gory (95% confidence interval 1.31, 2.41; p < .001).

Peak Level	LOS (days)	95% CI LL	UL	*P*
DELS17: 0	6.7	5.6	7.9	REF
DELS17: 1	7.4	6.6	8.3	.34
DELS17: 2 - 4	9.3	8.0	10.7	.004
DELS17: 5 - 6	9.8	7.7	11.9	.01
DELS17: 7 - 14	12.5	9.6	15.3	< .001
f2 = 0.042 , P = < .001				
Linear trend across categories	1.3	0.7	1.9	< .001

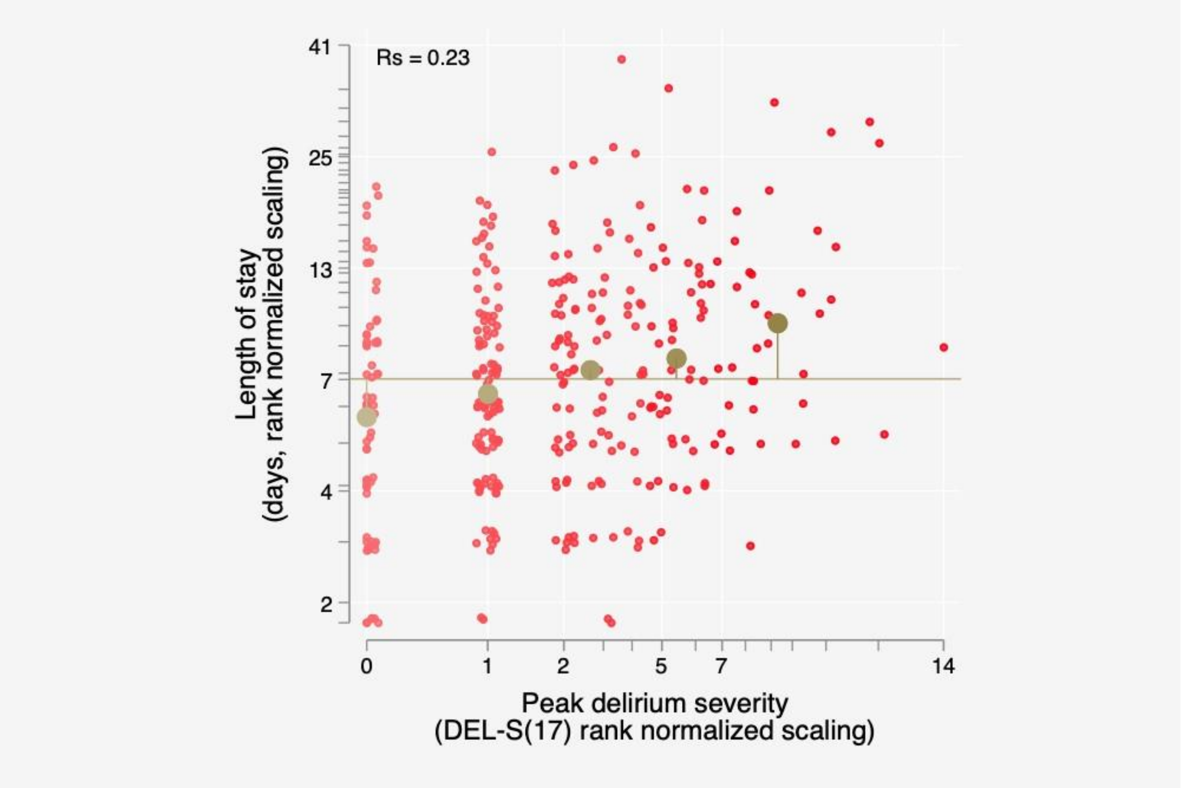

© 2022 Vasunilashorn SM et al. *JAMA Network Open.*

Length of stay - DELS Short Form

Differences across levels of delirium severity categories are tested with Poisson regression. Fold difference: The expected length of stay was 1.932 times as long for those in the highest severity category relative to the lowest severity cate- gory (95% confidence interval 1.32, 2.54; p < .001).

Peak Level	LOS (days)	95% CI LL	UL	*P*
DELS6: 0	6.9	5.8	7.9	REF
DELS6: 1	7.5	6.6	8.4	.37
DELS6: 2 - 3	9.5	8.0	11.1	.003
DELS6: 4 - 5	9.7	8.1	11.3	.004
DELS6: 6 - 9	13.3	9.8	16.7	< .001
f2 = 0.044 , P = < .001				
Linear trend across categories	1.4	0.8	2.0	< .001

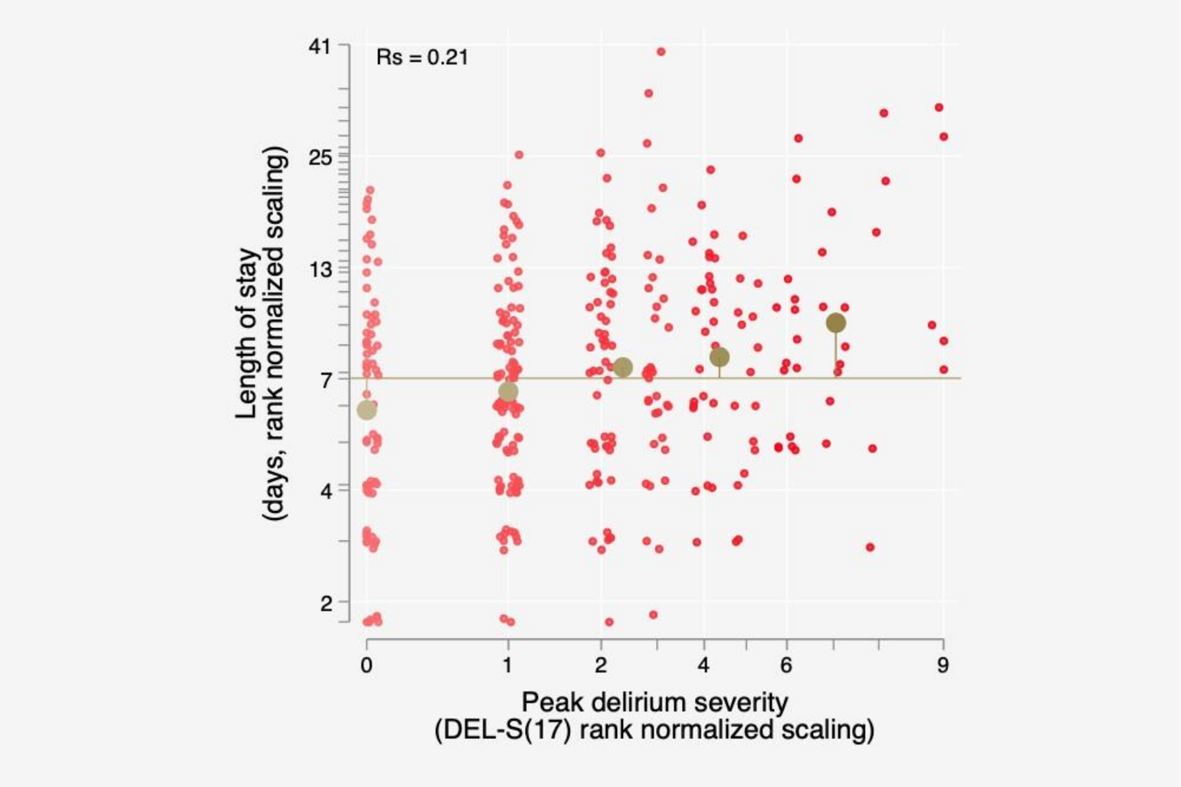

1.1.1 Missing data

This outcome has no missing data.

© 2022 Vasunilashorn SM et al. *JAMA Network Open.*

Regression model technical details

DELS long form

```
. poisson vdlos i.dels17cat cvdagebaseline cvdfemale cvdnonwhite
cvdcogimpair , vce(robust)Iteration 0:    log pseudolikelihood = -
1246.262
Iteration 1:   log pseudolikelihood = -1246.2617
Iteration 2:   log pseudolikelihood = -1246.2617
Poisson regression                              Number of obs     =        352
                                                Wald chi2(8)      =      34.38
                                                Prob > chi2       =     0.0000
Log pseudolikelihood = -1246.2617               Pseudo R2         =     0.0524
```

vdlos	Coef.	Robust Std. Err.	z	P>|z|	[95% Conf.	Interval]
dels17cat_peak						
DELS17: 1	.1028897	.107599	0.96	0.339	-.1080005	.3137798
DELS17: 2 - 4	.3277457	.11289	2.90	0.004	.1064853	.5490061
DELS17: 5 - 6	.3762319	.14685	2.56	0.010	.0884112	.6640525
DELS17: 7 - 14	.6201616	.1498899	4.14	0.000	.3263827	.9139404
cvdagebaseline	-.0049817	.0061442	-0.81	0.417	-.0170241	.0070606
cvdfemale	.0486041	.0788592	0.62	0.538	-.1059572	.2031653
cvdnonwhite	-.3505541	.1015694	-3.45	0.001	-.5496264	-.1514817
cvdcogimpair	-.1212554	.1049626	-1.16	0.248	-.3269783	.0844675
_cons	1.895645	.0890676	21.28	0.000	1.721076	2.070214

```
. margins dels17cat
Predictive margins                              Number of obs     =        352
 Model VCE    : Robust
Expression   : Predicted number of events, predict()
```

	Margin	Delta-method Std. Err.	z	P>|z|	[95% Conf.	Interval]
dels17cat_peak						
DELS17: 0	6.714723	.5932705	11.32	0.000	5.551934	7.877512
DELS17: 1	7.442392	.4459345	16.69	0.000	6.568376	8.316408
DELS17: 2 - 4	9.318935	.6806207	13.69	0.000	7.984943	10.65293
DELS17: 5 - 6	9.781907	1.079187	9.06	0.000	7.666741	11.89707
DELS17: 7 - 14	12.4842	1.458303	8.56	0.000	9.625984	15.34242

© 2022 Vasunilashorn SM et al. *JAMA Network Open.*

DELS short form

```
. poisson vdlos i.dels6cat cvdagebaseline cvdfemale cvdnonwhite
cvdcogimpair , vce(robust)Iteration 0:     log pseudolikelihood = -
1243.4633
Iteration 1:   log pseudolikelihood = -1243.4626
Iteration 2:   log pseudolikelihood = -1243.4626
Poisson regression                              Number of obs     =        352
                                                Wald chi2(8)      =      33.69
                                                Prob > chi2       =     0.0000
Log pseudolikelihood = -1243.4626               Pseudo R2         =     0.0545

------------------------------------------------------------------------------
             |               Robust
       vdlos |      Coef.   Std. Err.      z    P>|z|     [95% Conf. Interval]
-------------+----------------------------------------------------------------
dels6cat_peak|
    DELS6: 1 |   .0878004   .0988345     0.89   0.374    -.1059116    .2815124
DELS6: 2 - 3 |   .3303438   .1127994     2.93   0.003     .1092611    .5514265
DELS6: 4 - 5 |   .3456917   .1193358     2.90   0.004     .1117979    .5795855
DELS6: 6 - 9 |   .6583271   .1600814     4.11   0.000     .3445733     .972081
             |
cvdagebaseline|  -.0045651   .0061065    -0.75   0.455    -.0165336    .0074033
   cvdfemale |   .0471484   .0779152     0.61   0.545    -.1055627    .1998595
 cvdnonwhite |  -.3583622   .1027884    -3.49   0.000    -.5598237   -.1569008
cvdcogimpair |  -.1581177   .1039431    -1.52   0.128    -.3618424    .0456071
       _cons |   1.915908   .0793944    24.13   0.000     1.760298    2.071518
------------------------------------------------------------------------------

. margins dels6cat
Predictive margins                              Number of obs     =        352
Model VCE    : Robust
Expression   : Predicted number of events, predict()

------------------------------------------------------------------------------
             |            Delta-method
             |     Margin   Std. Err.      z    P>|z|     [95% Conf. Interval]
-------------+----------------------------------------------------------------
dels6cat_peak|
    DELS6: 0 |    6.86016   .5387948    12.73   0.000     5.804142    7.916179
    DELS6: 1 |   7.489718   .4451779    16.82   0.000     6.617186    8.362251
DELS6: 2 - 3 |   9.545545   .7828818    12.19   0.000     8.011125    11.07997
DELS6: 4 - 5 |    9.69318    .808876    11.98   0.000     8.107812    11.27855
DELS6: 6 - 9 |    13.2508   1.776635     7.46   0.000      9.76866    16.73294
------------------------------------------------------------------------------
```

© 2022 Vasunilashorn SM et al. *JAMA Network Open.*

Total hospital costs:

Total Health Care Costs of Index Hospitalization Data note. See Gou et al (2020, submitted) for details on source and description of costs data.

Methods note. We use a generalized linear model with log link to characterize total costs as a function of peak DELS severity during the hospital stay. We include age, sex, race/ethnicity, and Dementia or MCI at time of enrollment as potentially confounding variables. Effects of percentile rank normalized delirium severity are expressed for every 2 SD difference, following the recommendations of Gelman (2008).

These analyses are adjusted for age, sex, race, and cognitive impairment.

Results: These analyses make use of 299 observations with evaluable cost data. We used inverse propensity weights to account for missingness.

For peak score during hospitalization, each 2 standard deviation difference was associated with significantly increased costs: 2019USD 8,100 (95% CI: 2,200 - 13,900), after accounting for the effect of sex, race/ethnicity, age, and baseline cognitive impairment (f2 = 0.001 P=.01).
Fold difference: The expected costs were 1.705 times as much for those in the highest severity category relative to the lowest severity category (95% confidence interval 0.86, 2.55).

For peak score during hospitalization, each 2 standard deviation difference was associated with significantly increased costs: 2019USD 8,900 (95% CI: 3,400 - 14,400), after accounting for the effect of sex, race/ethnicity, age, and baseline cognitive impairment (f2 = 0.001 P=.004).
Fold difference: The expected costs were 2.025 times as much for those in the high- est severity category relative to the lowest severity category (95% confidence interval 1.06, 2.99).

Table
Mean Total Costs of Index Hospitalization (2019USD)by peak DELS level during hospitalization

Long form				Short form			
Level	N	Mean	SD	Level	N	Mean	SD
DELS17: 0	50	36,000	6,300	DELS6: 0	59	34,200	5,800
DELS17: 1	87	38,700	4,800	DELS6: 1	90	38,700	4,700
DELS17: 2 - 4	93	40,800	4,600	DELS6: 2 - 3	85	41,200	4,800
DELS17: 5 - 6	37	37,400	7,300	DELS6: 4 - 5	37	36,400	7,100
DELS17: 7 - 14	32	51,700	7,700[*]	DELS6: 6 - 9	28	57,700	8,100[**]

© 2022 Vasunilashorn SM et al. *JAMA Network Open.*

Regression model technical details

DELS long form

```
. glm medparcost_0_2019d cvdagebaseline cvdfemale cvdnonwhite cvdcogimpair i.dels17cat_peak [aweigh
> t=ipw_medparcost_0_2019d] ,
link(log) nolog(sum of wgt is
351.999999165535)

Generalized linear models                         Number of obs   =        299
Optimization     : ML                             Residual df     =        290
                                 Scale parameter =   2.03e+09
 Deviance         = 5.89753e+11        (1/df) Deviance =   2.03e+09
 Pearson          = 5.89753e+11        (1/df) Pearson  =   2.03e+09

Variance function: V(u) = 1                       [Gaussian]
Link function    : g(u) = ln(u)                   [Log]

                                                  AIC             =    24.3006
Log likelihood   = -3623.94025                    BIC             =   5.90e+11
```

medparcost_0_2019d	Coef.	OIM Std. Err.	z	P>|z|	[95% Conf.	Interval]
cvdagebaseline	-.0305298	.0112484	-2.71	0.007	-.0525762	-.0084834
cvdfemale	-.0238034	.1327772	-0.18	0.858	-.2840418	.236435
cvdnonwhite	-.6279236	.2665347	-2.36	0.018	-1.150322	-.1055252
cvdcogimpair	-.1831198	.1834604	-1.00	0.318	-.5426957	.176456
dels17cat_peak						
DELS17: 1	.2191211	.2218416	0.99	0.323	-.2156804	.6539227
DELS17: 2 - 4	.2869817	.2135644	1.34	0.179	-.1315969	.7055603
DELS17: 5 - 6	.2182613	.262372	0.83	0.405	-.2959784	.7325011
DELS17: 7 - 14	.5333541	.2527832	2.11	0.035	.0379082	1.0288
_cons	10.3187	.1945387	53.04	0.000	9.937409	10.69999

```
. margins , over(dels17cat_peak)

Predictive margins                             Number of obs     =        299
Model VCE    : OIM

Expression   : Predicted mean
medparcost_0_2019d, predict()over  :
dels17cat_peak
```

	Margin	Delta-method Std. Err.	z	P>|z|	[95% Conf.	Interval]
dels17cat_peak						
DELS17: 0	36022.41	6317.27	5.70	0.000	23640.79	48404.03
DELS17: 1	38681.31	4794.965	8.07	0.000	29283.35	48079.27
DELS17: 2 - 4	40765.68	4620.51	8.82	0.000	31709.65	49821.72
DELS17: 5 - 6	37358.07	7275.217	5.13	0.000	23098.91	51617.23
DELS17: 7 - 14	51726.68	7737.097	6.69	0.000	36562.25	66891.11

© 2022 Vasunilashorn SM et al. *JAMA Network Open.*

DELS short form

```
. glm medparcost_0_2019d cvdagebaseline cvdfemale cvdnonwhite cvdcogimpair i.dels6cat_peak [aweight
> =ipw_medparcost_0_2019d] ,
link(log) nolog(sum of wgt
is 351.999999165535)

Generalized linear models                        Number of obs   =        299
Optimization     : ML                            Residual df     =        290
                                Scale parameter =   2.01e+09
 Deviance        = 5.82652e+11          (1/df) Deviance =   2.01e+09
 Pearson         = 5.82652e+11          (1/df) Pearson  =   2.01e+09

Variance function: V(u) = 1                      [Gaussian]
Link function    : g(u) = ln(u)                  [Log]
                                                 AIC             =   24.28849
Log likelihood   = -3622.129209                  BIC             =   5.83e+11
```

medparcost_0_2019d	Coef.	OIM Std. Err.	z	P>|z|	[95% Conf.	Interval]
cvdagebaseline	-.0293564	.0110633	-2.65	0.008	-.05104	-.0076728
cvdfemale	-.0208723	.1318685	-0.16	0.874	-.2793299	.2375853
cvdnonwhite	-.6416503	.2638672	-2.43	0.015	-1.158821	-.1244801
cvdcogimpair	-.2806122	.1879596	-1.49	0.135	-.6490062	.0877819
dels6cat_peak						
DELS6: 1	.2241353	.2116415	1.06	0.290	-.1906745	.6389451
DELS6: 2 - 3	.3424533	.2078044	1.65	0.099	-.0648358	.7497423
DELS6: 4 - 5	.3436732	.2657607	1.29	0.196	-.1772081	.8645546
DELS6: 6 - 9	.7053321	.242059	2.91	0.004	.2309051	1.179759
_cons	10.27238	.1862176	55.16	0.000	9.907397	10.63736

```
. margins , over(dels6cat_peak)

Predictive margins                               Number of obs     =        299
Model VCE    : OIM

Expression   : Predicted mean
medparcost_0_2019d, predict()over  :
dels6cat_peak
```

	Margin	Delta-method Std. Err.	z	P>|z|	[95% Conf.	Interval]
dels6cat_peak						
DELS6: 0	34229	5757.516	5.95	0.000	22944.47	45513.52
DELS6: 1	38685.73	4691.801	8.25	0.000	29489.97	47881.49
DELS6: 2 - 3	41184.28	4801.684	8.58	0.000	31773.16	50595.41
DELS6: 4 - 5	36436.79	7115.794	5.12	0.000	22490.09	50383.49
DELS6: 6 - 9	57747	8141.869	7.09	0.000	41789.23	73704.77

© 2022 Vasunilashorn SM et al. *JAMA Network Open.*

Cumulative healthcare costs to 1 year

Methods note. We use a generalized linear model with log link to characterize total costs as a function of peak DELS severity during the hospital stay. We include age, sex, race/ethnicity, and Dementia or MCI at time of enrollment as potentially confounding variables. Effects of percentile rank normalized delirium severity are expressed for every 2 SD difference, following the recommendations of Gelman (2008).

Results

These analyses make use of 311 observations with evaluable cost data. We used inverse propensity weights to account for missingness.

Longform: For peak score during hospitalization, each 2 standard deviation difference was associated with significantly increased costs over 1 year: 2019USD 17,000 (95% CI: 1,900 - 32,100), after ac- counting for the effect of sex, race/ethnicity, age, and baseline cognitive impairment (f2 = 0.001 P=.03). **Fold difference:** The expected costs were 1.665 times as much for those in the highest severity category relative to the lowest severity category (95% confidence interval 0.96, 2.37).

Short Form: For peak score during hospitalization, each 2 standard deviation difference was associated with significantly increased costs over 1 year: 2019USD 20,000 (95% CI: 5,300 - 34,700), after ac- counting for the effect of sex, race/ethnicity, age, and baseline cognitive impairment (f2 = 0.001 P=.01). **Fold difference:** The expected costs were 1.755 times as much for those in the high-est severity category relative to the lowest severity category (95% confidence interval 1.05, 2.46).

Table
Mean 1-year costs (2019USD)
by peak DELS level during hospitalization

Long form				Short form			
Level	N	Mean	SD	Level	N	Mean	SD
DELS17: 0	56	106,100	17,000	DELS6: 0	65	106,500	15,800
DELS17: 1	88	105,000	13,600	DELS6: 1	92	104,700	13,200
DELS17: 2 - 4	95	123,300	12,900	DELS6: 2 - 3	87	118,600	13,300
DELS17: 5 - 6	38	111,300	20,500	DELS6: 4 - 5	37	122,900	20,200
DELS17: 7 - 14	34	158,400	21,100*	DELS6: 6 - 9	30	168,700	22,300**

© 2022 Vasunilashorn SM et al. *JAMA Network Open.*

Regression model technical details

DELS long form

```
. glm `xvar´ cvdagebaseline cvdfemale cvdnonwhite cvdcogimpair i.dels17cat_peak [aweight=ipw_`xvar´
> ] ,  link(log)  nolog
(sum of wgt is 352.0000013113022)

Generalized linear models                          Number of obs   =       311
Optimization     : ML                              Residual df     =       302
                                Scale parameter =   1.66e+10
Deviance =  5.02307e+12                 (1/df) Deviance =   1.66e+10
Pearson  =  5.02307e+12                 (1/df) Pearson  =   1.66e+10

Variance function: V(u) = 1                        [Gaussian]
Link function    : g(u) = ln(u)                    [Log]

                                                   AIC             =   26.40102
Log likelihood   = -4096.359209                    BIC             =   5.02e+12
```

totalcost_365_2019d	Coef.	OIM Std. Err.	z	P>|z|	[95% Conf.	Interval]
cvdagebaseline	-.0409506	.0114207	-3.59	0.000	-.0633348	-.0185663
cvdfemale	-.129619	.127317	-1.02	0.309	-.3791557	.1199177
cvdnonwhite	.0329115	.153555	0.21	0.830	-.2680507	.3338737
cvdcogimpair	-.2937364	.172894	-1.70	0.089	-.6326024	.0451295
dels17cat_peak						
DELS17: 1	.074419	.2035909	0.37	0.715	-.3246117	.4734497
DELS17: 2 - 4	.237108	.1921958	1.23	0.217	-.1395889	.6138049
DELS17: 5 - 6	.222506	.2390418	0.93	0.352	-.2460073	.6910192
DELS17: 7 - 14	.5101119	.214725	2.38	0.018	.0892586	.9309652
_cons	11.43811	.1706095	67.04	0.000	11.10372	11.77249

```
. margins , over(dels17cat_peak)

Predictive margins                                 Number of obs     =       311
Model VCE    : OIM

Expression   : Predicted mean
totalcost_365_2019d, predict()over :
dels17cat_peak
```

	Margin	Delta-method Std. Err.	z	P>|z|	[95% Conf.	Interval]
dels17cat_peak						
DELS17: 0	106123	16982.82	6.25	0.000	72837.28	139408.7
DELS17: 1	104987.2	13570.4	7.74	0.000	78389.75	131584.7
DELS17: 2 - 4	123347.2	12931.34	9.54	0.000	98002.2	148692.1
DELS17: 5 - 6	111271.5	20499.2	5.43	0.000	71093.8	151449.2
DELS17: 7 - 14	158417.3	21113.96	7.50	0.000	117034.7	199799.9

© 2022 Vasunilashorn SM et al. *JAMA Network Open.*

DELS short form

```
. glm `xvar´ cvdagebaseline cvdfemale cvdnonwhite cvdcogimpair i.dels6cat_peak [aweight=ipw_`xvar´]
> , link(log) nolog
(sum of wgt is 352.0000013113022)

Generalized linear models                      Number of obs   =        311
Optimization     : ML                          Residual df     =        302
                              Scale parameter =   1.65e+10
Deviance = 4.97101e+12                  (1/df) Deviance =   1.65e+10
Pearson  = 4.97101e+12                  (1/df) Pearson  =   1.65e+10

Variance function: V(u) = 1                    [Gaussian]
Link function    : g(u) = ln(u)                [Log]

                                               AIC             =   26.39061
Log likelihood   = -4094.739243                BIC             =   4.97e+12
```

totalcost_365_2019d	Coef.	OIM Std. Err.	z	P>|z|	[95% Conf.	Interval]
cvdagebaseline	-.0427225	.0117118	-3.65	0.000	-.0656771	-.0197678
cvdfemale	-.1339412	.1247742	-1.07	0.283	-.3784941	.1106118
cvdnonwhite	.0369947	.1504382	0.25	0.806	-.2578589	.3318482
cvdcogimpair	-.3265006	.1687188	-1.94	0.053	-.6571834	.0041822
dels6cat_peak						
DELS6: 1	.0555315	.1897873	0.29	0.770	-.3164449	.4275079
DELS6: 2 - 3	.21055	.1854191	1.14	0.256	-.1528647	.5739647
DELS6: 4 - 5	.430921	.2237329	1.93	0.054	-.0075875	.8694295
DELS6: 6 - 9	.5624366	.2041643	2.75	0.006	.1622819	.9625914
_cons	11.42741	.1608292	71.05	0.000	11.11219	11.74263

```
. margins , over(dels6cat_peak)

Predictive margins                             Number of obs    =        311
Model VCE    : OIM

Expression   : Predicted mean
totalcost_365_2019d, predict()over :
dels6cat_peak
```

	Margin	Delta-method Std. Err.	z	P>|z|	[95% Conf.	Interval]
dels6cat_peak						
DELS6: 0	106532.7	15773.12	6.75	0.000	75617.96	137447.5
DELS6: 1	104672.6	13177.51	7.94	0.000	78845.17	130500.1
DELS6: 2 - 3	118613	13301.32	8.92	0.000	92542.94	144683.1
DELS6: 4 - 5	122859.1	20197.76	6.08	0.000	83272.19	162446
DELS6: 6 - 9	168665.8	22306.4	7.56	0.000	124946	212385.5

© 2022 Vasunilashorn SM et al. *JAMA Network Open.*

Cumulative healthcare costs to 30 days

Methods note. We use a generalized linear model with log link to characterize total costs as a function of peak DELS severity during the hospital stay. We include age, sex, race/ethnicity, and Dementia or MCI at time of enrollment as potentially confounding variables. Effects of percentile rank normalized delirium severity are expressed for every 2 SD difference, following the recommendations of Gelman (2008). These analyses are adjusted for age, sex, race, and cognitive impairment.

Results

These analyses make use of 308 observations with evaluable cost data. We used inverse propensity weights to account for missingness.

Long form: For peak score during hospitalization, each 2 standard deviation difference was associated with significantly increased costs over 1 year: 2019USD 2,100 (95% CI: 0 - 4,200), after accounting for the effect of sex, race/ethnicity, age, and baseline cognitive impairment (f2 = 0.001 P=.06). **Fold difference:** The expected costs were 2.021 times as much for those in the highest severity category relative to the lowest severity category (95% confidence interval 0.78, 3.26).

Short Form: For peak score during hospitalization, each 2 standard deviation difference was associated with significantly increased costs over 1 year: 2019USD 2,400 (95% CI: 400 - 4,400), after accounting for the effect of sex, race/ethnicity, age, and baseline cognitive impairment (f2 = 0.001 P=.03). **Fold difference**: The expected costs were 1.763 times as much for those in the highest severity category relative to the lowest severity category (95% confidence interval 0.62, 2.90).

Total cost, discharge to 30 days
by peak DELS level during hospitalization

Long form				Short form			
Level	N	Mean	SD	Level	N	Mean	SD
DELS17: 0	55	10,000	2,300	DELS6: 0	64	9,300	2,100
DELS17: 1	88	12,300	1,900	DELS6: 1	92	11,600	1,800
DELS17: 2 - 4	94	10,400	1,800	DELS6: 2 - 3	86	11,200	1,800
DELS17: 5 - 6	38	13,900	2,700	DELS6: 4 - 5	37	17,700	2,700**
DELS17: 7 - 14	33	17,000	2,900*	DELS6: 6 - 9	29	14,200	3,100

© 2022 Vasunilashorn SM et al. *JAMA Network Open.*

Regression model technical details

DELS long form

```
. glm `xvar´ cvdagebaseline cvdfemale cvdnonwhite cvdcogimpair i.dels17cat_peak [aweight=ipw_`xvar´
> ] ,  link(log)  nolog
(sum of wgt is 351.9999996423721)

Generalized linear models                          Number of obs   =        308
Optimization     : ML                              Residual df     =        299
                                 Scale parameter =   3.12e+08
 Deviance         =  9.31491e+10        (1/df) Deviance =   3.12e+08
 Pearson          =  9.31491e+10        (1/df) Pearson  =   3.12e+08

Variance function: V(u) = 1                        [Gaussian]
Link function    : g(u) = ln(u)                    [Log]

                                                   AIC             =   22.42369
Log likelihood   = -3444.247677                    BIC             =   9.31e+10
```

totalcost_at30days_2019d	Coef.	OIM Std. Err.	z	P>|z|	[95% Conf.	Interval]
cvdagebaseline	-.0282833	.0138063	-2.05	0.041	-.0553431	-.0012234
cvdfemale	.1720969	.1712343	1.01	0.315	-.1635161	.5077099
cvdnonwhite	-1.24509	.5807728	-2.14	0.032	-2.383383	-.1067959
cvdcogimpair	.0185476	.1918021	0.10	0.923	-.3573777	.3944729
dels17cat_peak						
DELS17: 1	.3793872	.2791387	1.36	0.174	-.1677145	.9264889
DELS17: 2 - 4	.2048819	.2912124	0.70	0.482	-.3658838	.7756477
DELS17: 5 - 6	.4518062	.3166877	1.43	0.154	-.1688903	1.072503
DELS17: 7 - 14	.7038118	.3119274	2.26	0.024	.0924453	1.315178
_cons	8.996861	.2595806	34.66	0.000	8.488092	9.50563

```
. margins , over(dels17cat_peak)

Predictive margins                              Number of obs     =        308
Model VCE    : OIM

Expression   : Predicted  mean
totalcost_at30days_2019d, predict()over     :
dels17cat_peak
```

	Margin	Delta-method Std. Err.	z	P>|z|	[95% Conf.	Interval]
dels17cat_peak						
DELS17: 0	9988.801	2315.221	4.31	0.000	5451.052	14526.55
DELS17: 1	12288.73	1852.223	6.63	0.000	8658.436	15919.02
DELS17: 2 - 4	10407.98	1770.614	5.88	0.000	6937.64	13878.32
DELS17: 5 - 6	13873.61	2729.026	5.08	0.000	8524.822	19222.41
DELS17: 7 - 14	16978.54	2922.837	5.81	0.000	11249.88	22707.19

© 2022 Vasunilashorn SM et al. *JAMA Network Open.*

DELS short form

```
. glm `xvar´ cvdagebaseline cvdfemale cvdnonwhite cvdcogimpair i.dels6cat_peak [aweight=ipw_`xvar´]
>  , link(log) nolog
(sum of wgt is 351.9999996423721)

Generalized linear models                       Number of obs   =        308
Optimization     : ML                           Residual df     =        299
                              Scale parameter =   3.08e+08
Deviance =  9.20953e+10               (1/df) Deviance =   3.08e+08
Pearson  =  9.20953e+10               (1/df) Pearson  =   3.08e+08

Variance function: V(u) = 1                     [Gaussian]
Link function    : g(u) = ln(u)                 [Log]

                                                AIC             =   22.41231
Log likelihood   = -3442.495555                 BIC             =   9.21e+10
```

totalcost_at30days_2019d	Coef.	OIM Std. Err.	z	P>|z|	[95% Conf.	Interval]
cvdagebaseline	-.0328607	.0142313	-2.31	0.021	-.0607535	-.0049679
cvdfemale	.1083654	.1616049	0.67	0.503	-.2083743	.4251051
cvdnonwhite	-1.277352	.5804579	-2.20	0.028	-2.415029	-.1396757
cvdcogimpair	-.0559861	.1913373	-0.29	0.770	-.4310004	.3190281
dels6cat_peak						
DELS6: 1	.336286	.2753529	1.22	0.222	-.2033958	.8759677
DELS6: 2 - 3	.3255579	.2865136	1.14	0.256	-.2359986	.8871143
DELS6: 4 - 5	.8750425	.2943414	2.97	0.003	.2981439	1.451941
DELS6: 6 - 9	.5667833	.3299207	1.72	0.086	-.0798493	1.213416
_cons	8.935603	.2547627	35.07	0.000	8.436277	9.434929

```
. margins , over(dels6cat_peak)

Predictive margins                            Number of obs     =        308
Model VCE    : OIM

Expression   : Predicted mean
totalcost_at30days_2019d, predict()over     :
dels6cat_peak
```

	Margin	Delta-method Std. Err.	z	P>|z|	[95% Conf.	Interval]
dels6cat_peak						
DELS6: 0	9313.14	2123.868	4.38	0.000	5150.435	13475.84
DELS6: 1	11579.35	1811.972	6.39	0.000	8027.955	15130.75
DELS6: 2 - 3	11170.24	1817.636	6.15	0.000	7607.734	14732.74
DELS6: 4 - 5	17723.94	2723.084	6.51	0.000	12386.79	23061.08
DELS6: 6 - 9	14191.69	3060.653	4.64	0.000	8192.92	20190.46

© 2022 Vasunilashorn SM et al. *JAMA Network Open.*

Rehospitalization within 90 days

Methods note. We use Poisson regression with robust variance estimation to characterize the risk of Any rehospitalization within 90 days as a function of of peak DELS severity during the hospital stay. We include age, sex, race/ethnicity, and Dementia or MCI at time of enrollment as potentially confounding variables. Effects of percentile rank normalized delirium severity are expressed for every 2 SD difference, following the recommendations of Gelman (2008).

Results

These analyses made use of 352 participants. Of these, 119 (34%) participants had Any rehospitalization within 90 days. We used inverse propensity weights to account for missingness.

Long Form: For DELS (long form) peak score during hospitalization, each 2 standard deviation difference was associated with significantly increased risk of Any rehospitalization within 90 days: relative risk 1.09 (95% Confidence Interval: 0.80 - 1.49, P = .58). **Fold difference**: The expected costs were 1.217 times as much for those in the highest severity category relative to the lowest severity category (95% confidence interval 0.38, 2.06).

Short Form: For DELS (short form) peak score during hospitalization, each 2 standard deviation difference was associated with significantly increased risk of Any rehospitalization within 90 days: relative risk 1.09 (95% Confidence Interval: 0.80 - 1.49, P = .57).
Fold difference: The expected costs were 1.035 times as much for those in the highest severity category relative to the lowest severity category (95% confidence interval 0.27, 1.80).

Any rehospitalization within 90 days by peak DELS level during hospitalization

Level	n/N	Percent
Short Form		
DELS6: 0	22/63	35[na]
DELS6: 1	30/93	32[ns]
DELS6: 2 - 3	37/88	42[ns]
DELS6: 4 - 5	18/37	49[ns]
DELS6: 6 - 9	12/30	40[ns]
Long form		
DELS17: 0	20/53	38[na]
DELS17: 1	27/90	30[ns]
DELS17: 2 - 4	41/96	43[ns]
DELS17: 5 - 6	15/39	38[ns]
DELS17: 7 - 14	16/33	48[ns]

© 2022 Vasunilashorn SM et al. *JAMA Network Open.*

Regression model technical details

DELS long form

```
. glm `y´ cvdagebaseline cvdfemale cvdnonwhite cvdcogimpair i.dels17cat_peak [aweight=ipw_`y´],  fa
> m(poisson)
link(log)  nolog
(sum of wgt is
351.999999761581
4)

Generalized linear models                          Number of obs   =       311
Optimization     : ML                              Residual df     =       302
                                                   Scale parameter =         1
Deviance         =  221.7516491                    (1/df) Deviance =   .734277
Pearson          =  191.9752142                    (1/df) Pearson  =  .6356795

Variance function: V(u) = u                        [Poisson]
Link function    : g(u) = ln(u)                    [Log]

                                                   AIC             =   1.53607
Log likelihood   = -229.8589314                    BIC             =  -1511.666
```

rehosp_any_90	Coef.	OIM Std. Err.	z	P>|z|	[95% Conf.	Interval]
cvdagebaseline	-.0140508	.0143968	-0.98	0.329	-.0422679	.0141663
cvdfemale	.0814146	.1896045	0.43	0.668	-.2902034	.4530326
cvdnonwhite	-.3402133	.2941154	-1.16	0.247	-.9166688	.2362422
cvdcogimpair	.3021335	.2141279	1.41	0.158	-.1175494	.7218165
dels17cat_peak						
DELS17: 1	-.1780071	.3003774	-0.59	0.553	-.766736	.4107219
DELS17: 2 - 4	.120485	.2809115	0.43	0.668	-.4300915	.6710614
DELS17: 5 - 6	-.0521684	.3552283	-0.15	0.883	-.748403	.6440662
DELS17: 7 - 14	.1964993	.3521991	0.56	0.577	-.4937983	.8867969
_cons	-.9898967	.2310507	-4.28	0.000	-1.442748	-.5370456

```
. margins , over(dels17cat_peak)

Predictive margins                              Number of obs     =        311
Model VCE    : OIM

Expression   : Predicted mean
rehosp_any_90, predict()over       :
dels17cat_peak
```

	Margin	Delta-method Std. Err.	z	P>|z|	[95% Conf.	Interval]
dels17cat_peak						
DELS17: 0	.3794866	.0845789	4.49	0.000	.2137151	.5452581
DELS17: 1	.2996242	.0578129	5.18	0.000	.186313	.4129354
DELS17: 2 - 4	.4271901	.0667588	6.40	0.000	.2963453	.5580349
DELS17: 5 - 6	.3829984	.0987113	3.88	0.000	.1895278	.5764691
DELS17: 7 - 14	.4824071	.1206379	4.00	0.000	.245961	.7188531

© 2022 Vasunilashorn SM et al. *JAMA Network Open.*

DELS short form

```
. glm `y´ cvdagebaseline cvdfemale cvdnonwhite cvdcogimpair i.dels6cat_peak [aweight=ipw_`y´],  fam
> (poisson)
link(log)  nolog
(sum of wgt is
351.999999761581
4)

Generalized linear models                         Number of obs   =       311
Optimization    : ML                              Residual df     =       302
                                                  Scale parameter =         1
Deviance        = 222.0642808                     (1/df) Deviance =  .7353122
Pearson         = 192.2194392                     (1/df) Pearson  =  .6364882

Variance function: V(u) = u                       [Poisson]
Link function   : g(u) = ln(u)                    [Log]

                                                  AIC             =  1.537076
Log likelihood  = -230.0152473                    BIC             = -1511.353
```

rehosp_any_90	Coef.	OIM Std. Err.	z	P>|z|	[95% Conf.	Interval]
cvdagebaseline	-.0175474	.0145754	-1.20	0.229	-.0461146	.0110198
cvdfemale	.0693083	.1887983	0.37	0.714	-.3007296	.4393462
cvdnonwhite	-.3568388	.2929396	-1.22	0.223	-.9309899	.2173123
cvdcogimpair	.2866179	.2184268	1.31	0.189	-.1414908	.7147266
dels6cat_peak						
DELS6: 1	-.048499	.2846686	-0.17	0.865	-.6064393	.5094413
DELS6: 2 - 3	.1746136	.2768152	0.63	0.528	-.3679341	.7171613
DELS6: 4 - 5	.3023544	.3413546	0.89	0.376	-.3666884	.9713971
DELS6: 6 - 9	.0347021	.3791307	0.09	0.927	-.7083804	.7777846
_cons	-1.06252	.2207077	-4.81	0.000	-1.4951	-.6299412

```
. margins , over(dels6cat_peak)

Predictive margins                              Number of obs     =       311
Model VCE    : OIM

Expression   : Predicted mean
rehosp_any_90, predict()over      :
dels6cat_peak
```

	Margin	Delta-method Std. Err.	z	P>|z|	[95% Conf.	Interval]
dels6cat_peak						
DELS6: 0	.3508436	.0745431	4.71	0.000	.2047418	.4969455
DELS6: 1	.3222846	.0589987	5.46	0.000	.2066492	.43792
DELS6: 2 - 3	.4202964	.0690994	6.08	0.000	.284864	.5557288
DELS6: 4 - 5	.4859652	.1144815	4.24	0.000	.2615855	.7103449
DELS6: 6 - 9	.3969556	.1147084	3.46	0.001	.1721313	.6217799

© 2022 Vasunilashorn SM et al. *JAMA Network Open.*

Rehospitalization within 1 year

Methods note. We use Poisson regression with robust variance estimation to characterize the riskof Any rehospitalization within 365 days as a function of peak DELS severity during the hospitalstay. We include age, sex, race/ethnicity, and Dementia or MCI at time of enrollment as potentiallyconfounding variables. Effects of percentile rank normalized delirium severity are expressed for every 2 SD difference, following the recommendations of Gelman (2008).

Results
These analyses made use of 352 participants. Of these, 197 (56%) participants had Any rehospitalization within 365 days. We used inverse propensity weights to account for missingness.

Long Form: For DELS (long form) peak score during hospitalization, each 2 standard deviation difference wasassociated with significantly increased risk of Any rehospitalization within 365 days: relative risk 1.12 (95% Confidence Interval: 0.94 - 1.34, P = .19). **Fold difference:** The expected costs were 1.286 times as much for those in the highest severity category relative to the lowest severity category (95% confidence interval 0.60, 1.97).

Short Form: For DELS (short form) peak score during hospitalization, each 2 standard deviation difference was associated with significantly increased risk of Any rehospitalization within 365 days: relative risk 1.12 (95% Confidence Interval: 0.94 - 1.35, P = .21). **Fold difference**: The expected costs were 1.231mes as much for those in the highest severity category relative to the lowest severity category (95% confidence interval 0.56, 1.90).

Any rehospitalization within 365 days by peak DELS level during hospitalization

Level	n/N	Percent
	Short form	
DELS6: 0	38/63	61[na]
DELS6: 1	53/93	57[ns]
DELS6: 2 - 3	55/88	62[ns]
DELS6: 4 - 5	28/37	76[ns]
DELS6: 6 - 9	23/30	76[ns]
	Long form	
DELS17: 0	33/53	63[na]
DELS17: 1	51/90	57[ns]
DELS17: 2 - 4	59/96	61[ns]
DELS17: 5 - 6	27/39	69[ns]
DELS17: 7 - 14	27/33	82[ns]

© 2022 Vasunilashorn SM et al. *JAMA Network Open.*

Regression model technical details

DELS long form

```
. glm `y´ cvdagebaseline cvdfemale cvdnonwhite cvdcogimpair i.dels17cat_peak [aweight=ipw_`y´],  fa
> m(poisson)
link(log)  nolog
(sum of wgt is
352.000001192092
9)

Generalized linear models                      Number of obs   =        311
Optimization    : ML                           Residual df     =        302
                                               Scale parameter =          1
Deviance        = 175.2423718                  (1/df) Deviance =   .5802728
Pearson         = 113.8444233                  (1/df) Pearson  =   .3769683

Variance function: V(u) = u                    [Poisson]
Link function    : g(u) = ln(u)                [Log]

                                               AIC             =   1.888023
Log likelihood   = -284.5875509                BIC             =  -1558.175
```

rehosp_any_365	Coef.	OIM Std. Err.	z	P>|z|	[95% Conf.	Interval]
cvdagebaseline	-.0070207	.0110152	-0.64	0.524	-.0286102	.0145688
cvdfemale	-.0503605	.1461767	-0.34	0.730	-.3368615	.2361406
cvdnonwhite	-.2586108	.2254593	-1.15	0.251	-.700503	.1832814
cvdcogimpair	.1002906	.1724215	0.58	0.561	-.2376494	.4382305
dels17cat_peak						
DELS17: 1	-.0658724	.2272369	-0.29	0.772	-.5112486	.3795038
DELS17: 2 - 4	-.000793	.2231885	-0.00	0.997	-.4382346	.4366485
DELS17: 5 - 6	.0879256	.269712	0.33	0.744	-.4407001	.6165513
DELS17: 7 - 14	.251889	.2712052	0.93	0.353	-.2796634	.7834413
_cons	-.4871908	.1793149	-2.72	0.007	-.8386416	-.13574

```
. margins , over(dels17cat_peak)

Predictive margins                             Number of obs     =        311
Model VCE    : OIM

Expression   : Predicted mean
rehosp_any_365, predict()over      :
dels17cat_peak
```

	Margin	Delta-method Std. Err.	z	P>|z|	[95% Conf.	Interval]
dels17cat_peak						
DELS17: 0	.6256689	.1085956	5.76	0.000	.4128255	.8385124
DELS17: 1	.5664331	.0794915	7.13	0.000	.4106327	.7222336
DELS17: 2 - 4	.6136728	.0800108	7.67	0.000	.4568544	.7704912
DELS17: 5 - 6	.687741	.1322858	5.20	0.000	.4284657	.9470163
DELS17: 7 - 14	.8188819	.1571848	5.21	0.000	.5108053	1.126958

© 2022 Vasunilashorn SM et al. *JAMA Network Open.*

DELS short form

```
. glm `y´ cvdagebaseline cvdfemale cvdnonwhite cvdcogimpair i.dels6cat_peak [aweight=ipw_`y´],   fam
> (poisson)
link(log)  nolog
(sum of wgt is
352.000001192092
9)

Generalized linear models                    Number of obs   =       311
Optimization    : ML                         Residual df     =       302
                                             Scale parameter =         1
Deviance        =  175.248612                (1/df) Deviance =  .5802934
Pearson         = 114.0185459                (1/df) Pearson  =  .3775449

Variance function: V(u) = u                  [Poisson]
Link function    : g(u) = ln(u)              [Log]

                                             AIC             =  1.888043
Log likelihood   = -284.590671               BIC             = -1558.169
```

rehosp_any_365	Coef.	OIM Std. Err.	z	P>|z|	[95% Conf.	Interval]
cvdagebaseline	-.0084183	.0111055	-0.76	0.448	-.0301847	.013348
cvdfemale	-.0580931	.145623	-0.40	0.690	-.3435089	.2273227
cvdnonwhite	-.2751774	.2244931	-1.23	0.220	-.7151757	.1648209
cvdcogimpair	.0756659	.1760897	0.43	0.667	-.2694635	.4207954
dels6cat_peak						
DELS6: 1	-.0454806	.2153365	-0.21	0.833	-.4675324	.3765711
DELS6: 2 - 3	.0412779	.216189	0.19	0.849	-.3824449	.4650006
DELS6: 4 - 5	.2330889	.2662423	0.88	0.381	-.2887364	.7549143
DELS6: 6 - 9	.2075197	.2794278	0.74	0.458	-.3401487	.7551881
_cons	-.5146395	.1675461	-3.07	0.002	-.8430239	-.1862552

```
. margins , over(dels6cat_peak)

Predictive margins                          Number of obs     =       311
Model VCE    : OIM

Expression   : Predicted mean
rehosp_any_365, predict()over      :
dels6cat_peak
```

	Margin	Delta-method Std. Err.	z	P>|z|	[95% Conf.	Interval]
dels6cat_peak						
DELS6: 0	.6057442	.0979445	6.18	0.000	.4137764	.7977119
DELS6: 1	.569544	.0784324	7.26	0.000	.4158193	.7232686
DELS6: 2 - 3	.6238023	.0841794	7.41	0.000	.4588137	.788791
DELS6: 4 - 5	.7554049	.1427388	5.29	0.000	.4756421	1.035168
DELS6: 6 - 9	.7646342	.1592112	4.80	0.000	.452586	1.076682

© 2022 Vasunilashorn SM et al. *JAMA Network Open.*

Deaths at 30 days

Methods note. We use Poisson regression with robust variance estimation to characterize the risk of death within 30 days as a function of peak DELS severity during the hospital stay. We include age, sex, race/ethnicity, and Dementia or MCI at time of enrollment as potentially confounding variables. Effects of percentile rank normalized delirium severity are expressed for every 2 SD difference, following the recommendations of Gelman (2008).

Results

These analyses made use of 352 participants. Of these, 20 (6%) participants died by the one month follow-up.

Long Form: For DELS (long form) peak score during hospitalization, each 2 standard deviation difference was associated with significantly increased risk of death by 30 days: relative risk 1.63 (95% Confidence Interval: 0.54 - 4.94, P = .39). The risk of death was 2.25 for those in the highest severity category relative to the lowest severity category (95% confidence interval 0.52, 13.13). (NB: confidence intervals obtained with bootstrap resampling.)

Short Form: For DELS (short form) peak score during hospitalization, each 2 standard deviation difference was associated with significantly increased risk of death by 30 days: relative risk 1.59 (95% Confidence Interval: 0.51 - 4.92, P = .42). The risk of death was 1.60 for those in the highest severity category relative to the lowest severity category (95% confidence interval 0.28, 11.59). (NB: confidence intervals obtained with bootstrap resampling.)

Table
Deaths at one-month interview
by peak DELS level during hospitalization

Long form			Short form		
Level	n/N	Percent	Level	n/N	Percent
DELS17: 0	4.0/64	6.0[na]	DELS6: 0	5.0/75	7.0[na]
DELS17: 1	5.0/104	5.0	DELS6: 1	4.0/108	4.0
DELS17: 2 - 4	3.0/101	3.0	DELS6: 2 - 3	4.0/91	4.0
DELS17: 5 - 6	2.0/44	5.0	DELS6: 4 - 5	3.0/44	7.0
DELS17: 7 - 14	6.0/39	15.0	DELS6: 6 - 9	4.0/34	12.0

© 2022 Vasunilashorn SM et al. *JAMA Network Open.*

Regression model technical details

DELS long form

```
. logit dead30 cvdagebaseline cvdfemale cvdnonwhite cvdcogimpair
i.dels17cat_peak , nologLogistic regression     Number of obs     =        352
                                                LR chi2(8)        =      14.22
                                                Prob > chi2       =     0.0762
Log likelihood = -69.668733                     Pseudo R2         =     0.0926
```

dead30	Coef.	Std. Err.	z	P>|z|	[95% Conf.	Interval]
cvdagebaseline	.0777431	.0349399	2.23	0.026	.0092622	.1462241
cvdfemale	.1605848	.4904175	0.33	0.743	-.8006158	1.121785
cvdnonwhite	-.9225825	1.052775	-0.88	0.381	-2.985983	1.140818
cvdcogimpair	.2275719	.5596406	0.41	0.684	-.8693035	1.324447
dels17cat_peak						
DELS17: 1	-.4467331	.7070004	-0.63	0.527	-1.832428	.9389623
DELS17: 2 - 4	-.9079293	.8005301	-1.13	0.257	-2.476939	.6610808
DELS17: 5 - 6	-.6242502	.9404031	-0.66	0.507	-2.467406	1.218906
DELS17: 7 - 14	.8247811	.7476556	1.10	0.270	-.6405969	2.290159
_cons	-2.757897	.5434016	-5.08	0.000	-3.822945	-1.69285

```
. margins , over(dels17cat_peak)

Predictive margins                              Number of obs     =        352
Model VCE    : OIM

Expression   :
Pr(dead30),
predict()over     :
dels17cat_peak
```

	Delta-method					
	Margin	Std. Err.	z	P>|z|	[95% Conf.	Interval]
dels17cat_peak						
DELS17: 0	.0625	.0299178	2.09	0.037	.0038622	.1211378
DELS17: 1	.0480769	.0207764	2.31	0.021	.0073559	.088798
DELS17: 2 - 4	.029703	.0167855	1.77	0.077	-.0031959	.0626019
DELS17: 5 - 6	.0454545	.0311182	1.46	0.144	-.015536	.1064451
DELS17: 7 - 14	.1538462	.0559514	2.75	0.006	.0441834	.2635089

© 2022 Vasunilashorn SM et al. *JAMA Network Open.*

DELS short form

```
. logit dead30 cvdagebaseline cvdfemale cvdnonwhite cvdcogimpair
i.dels6cat_peak , nologLogistic regression     Number of obs     =        352
                                               LR chi2(8)        =      10.81
                                               Prob > chi2       =     0.2128
Log likelihood = -71.374271                    Pseudo R2         =     0.0704
```

dead30	Coef.	Std. Err.	z	P>|z|	[95% Conf.	Interval]
cvdagebaseline	.0783873	.0345017	2.27	0.023	.0107652	.1460094
cvdfemale	.0632097	.4843729	0.13	0.896	-.8861438	1.012563
cvdnonwhite	-.9860373	1.050026	-0.94	0.348	-3.044051	1.071976
cvdcogimpair	.1975271	.5821507	0.34	0.734	-.9434674	1.338522
dels6cat_peak						
DELS6: 1	-.736483	.7032496	-1.05	0.295	-2.114827	.6418609
DELS6: 2 - 3	-.544904	.7096474	-0.77	0.443	-1.935787	.8459793
DELS6: 4 - 5	-.3595793	.830613	-0.43	0.665	-1.987551	1.268392
DELS6: 6 - 9	.4996306	.8158731	0.61	0.540	-1.099451	2.098713
_cons	-2.710336	.4983174	-5.44	0.000	-3.68702	-1.733652

```
. margins , over(dels6cat_peak)
Predictive margins                             Number of obs     =        352
Model VCE    : OIM
Expression   :
Pr(dead30),
predict()over     :
dels6cat_peak
```

	Margin	Delta-method Std. Err.	z	P>|z|	[95% Conf.	Interval]
dels6cat_peak						
DELS6: 0	.0666667	.0284228	2.35	0.019	.010959	.1223744
DELS6: 1	.037037	.0180425	2.05	0.040	.0016744	.0723997
DELS6: 2 - 3	.043956	.0213106	2.06	0.039	.002188	.0857241
DELS6: 4 - 5	.0681818	.0374826	1.82	0.069	-.0052828	.1416464
DELS6: 6 - 9	.1176471	.0537824	2.19	0.029	.0122356	.2230585

© 2022 Vasunilashorn SM et al. *JAMA Network Open.*

Deaths at 1 year

Methods note. We use Poisson regression with robust variance estimation to characterize the risk of death within 30 days as a function of peak DELS severity during the hospital stay. We include age, sex, race/ethnicity, and Dementia or MCI at time of enrollment as potentially confounding variables. Effects of percentile rank normalized delirium severity are expressed for every 2 SD difference, following the recommendations of Gelman (2008).

Results
These analyses made use of 352 participants. Of these, 92 (26.1%) participants died by one year follow-up.

Long Form: For DELS (long form) peak score during hospitalization, each 2 standard deviation difference was associated with significantly increased risk of death by 365 days: relative risk 1.48 (95% Confidence Interval: 1.03 - 2.11, P = .03). The risk of death was 2.740 for those in the highest severity category relative to the lowest severity category (95% confidence interval 0.13, 5.35; P = .04).

Short Form: For DELS (short form) peak score during hospitalization, each 2 standard deviation difference was associated with significantly increased risk of death by 365 days: relative risk 1.55 (95% Confidence Interval: 1.08 - 2.22, P = .02). The risk of death was 3.666 for those in the highest severity category relative to the lowest severity category (95% confidence interval 0.06, 7.28; P = .046).

Table
Deaths at one year interview
by peak DELS level during hospitalization

Long form			Short form		
Level	n/N	Percent	Level	n/N	Percent
DELS17: 0	12/64	19.0[na]	DELS6: 0	13/75	17.0[na]
DELS17: 1	22/104	21.0	DELS6: 1	23/108	21.0
DELS17: 2 - 4	28/101	28.0	DELS6: 2 - 3	27/91	30.0
DELS17: 5 - 6	12/44	27.0	DELS6: 4 - 5	12/44	27.0
DELS17: 7 - 14	18/39	46.0[*]	DELS6: 6 - 9	17/34	50.0[**]

© 2022 Vasunilashorn SM et al. *JAMA Network Open.*

Regression model technical details

DELS long form

```
. logit dead365 cvdagebaseline cvdfemale cvdnonwhite cvdcogimpair
i.dels17cat_peak , nologLogistic regression     Number of obs     =        352
                                                LR chi2(8)        =      28.50
                                                Prob > chi2       =     0.0004
Log likelihood = -187.96459                     Pseudo R2         =     0.0705
```

dead365	Coef.	Std. Err.	z	P>|z|	[95% Conf.	Interval]
cvdagebaseline	.0552443	.0190835	2.89	0.004	.0178413	.0926473
cvdfemale	.0803988	.2608294	0.31	0.758	-.4308175	.5916151
cvdnonwhite	-.3053751	.3945445	-0.77	0.439	-1.078668	.4679179
cvdcogimpair	.7042429	.2929409	2.40	0.016	.1300893	1.278396
dels17cat_peak						
DELS17: 1	.0095819	.4130395	0.02	0.981	-.7999607	.8191245
DELS17: 2 - 4	.3237795	.4060228	0.80	0.425	-.4720105	1.119569
DELS17: 5 - 6	.0594956	.4987032	0.12	0.905	-.9179447	1.036936
DELS17: 7 - 14	1.007836	.4854421	2.08	0.038	.0563872	1.959285
_cons	-1.344046	.3310118	-4.06	0.000	-1.992817	-.6952749

```
. margins , over(dels17cat_peak)
Predictive margins                              Number of obs     =        352
Model VCE    : OIM
Expression   :
Pr(dead365),
predict()over    :
dels17cat_peak
```

	Delta-method Margin	Std. Err.	z	P>|z|	[95% Conf.	Interval]
dels17cat_peak						
DELS17: 0	.1875	.0478802	3.92	0.000	.0936565	.2813435
DELS17: 1	.2115385	.0391661	5.40	0.000	.1347743	.2883026
DELS17: 2 - 4	.2772277	.043399	6.39	0.000	.1921672	.3622883
DELS17: 5 - 6	.2727273	.0649631	4.20	0.000	.145402	.4000526
DELS17: 7 - 14	.4615385	.0764344	6.04	0.000	.3117297	.6113472

© 2022 Vasunilashorn SM et al. *JAMA Network Open.*

DELS short form

```
. logit dead365 cvdagebaseline cvdfemale cvdnonwhite cvdcogimpair
i.dels6cat_peak , nologLogistic regression      Number of obs     =        352
                                                LR chi2(8)        =      31.22
                                                Prob > chi2       =     0.0001
Log likelihood = -186.60715                     Pseudo R2         =     0.0772
```

dead365	Coef.	Std. Err.	z	P>|z|	[95% Conf.	Interval]
cvdagebaseline	.0589548	.0193587	3.05	0.002	.0210124	.0968972
cvdfemale	.0668353	.2613701	0.26	0.798	-.4454407	.5791113
cvdnonwhite	-.3142353	.3944674	-0.80	0.426	-1.087377	.4589065
cvdcogimpair	.6294452	.3004832	2.09	0.036	.0405089	1.218381
dels6cat_peak						
DELS6: 1	.1477922	.3963828	0.37	0.709	-.6291038	.9246882
DELS6: 2 - 3	.5389132	.3970569	1.36	0.175	-.2393041	1.317131
DELS6: 4 - 5	.0856022	.4955211	0.17	0.863	-.8856014	1.056806
DELS6: 6 - 9	1.299023	.5026058	2.58	0.010	.3139337	2.284112
_cons	-1.45868	.3164683	-4.61	0.000	-2.078947	-.8384139

```
. margins , over(dels6cat_peak)
Predictive margins                              Number of obs     =        352
Model VCE    : OIM
Expression   :
Pr(dead365),
predict()over     :
dels6cat_peak
```

	Margin	Delta-method Std. Err.	z	P>|z|	[95% Conf.	Interval]
dels6cat_peak						
DELS6: 0	.1733333	.0428994	4.04	0.000	.089252	.2574147
DELS6: 1	.212963	.0385397	5.53	0.000	.1374265	.2884994
DELS6: 2 - 3	.2967033	.0467592	6.35	0.000	.2050569	.3883497
DELS6: 4 - 5	.2727273	.0650718	4.19	0.000	.1451889	.4002656
DELS6: 6 - 9	.5	.0818328	6.11	0.000	.3396107	.6603893

© 2022 Vasunilashorn SM et al. *JAMA Network Open.*

eFigure 1. Breakdown of the Available Study Data

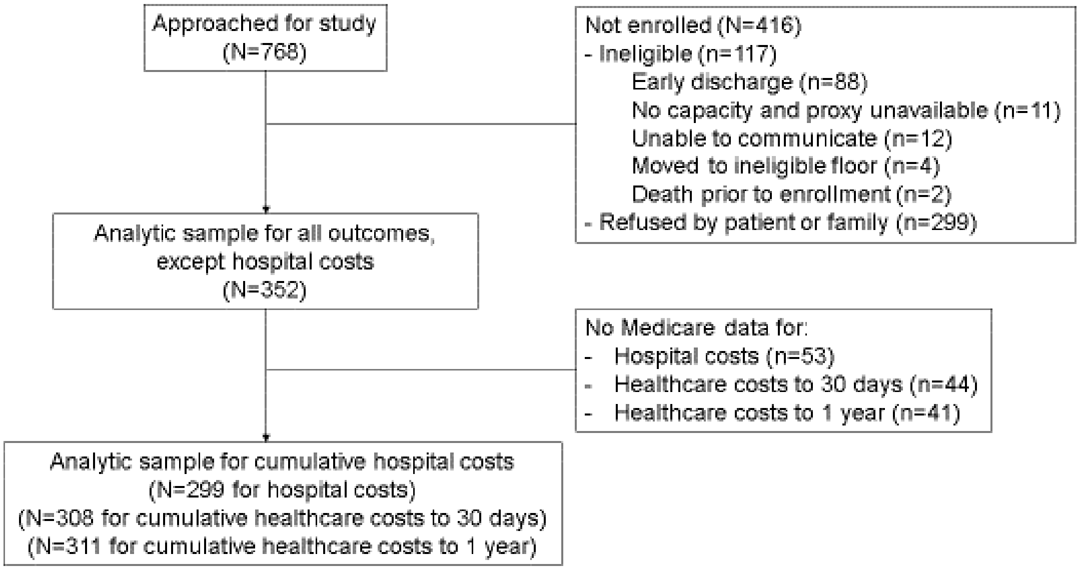

© 2022 Vasunilashorn SM et al. *JAMA Network Open.*

eFigure 2. Histograms of DEL-S

eFigure 2A
Histogram of DELS-SF among 1,190 assessments conducted
in 352 patients duringhospitalization

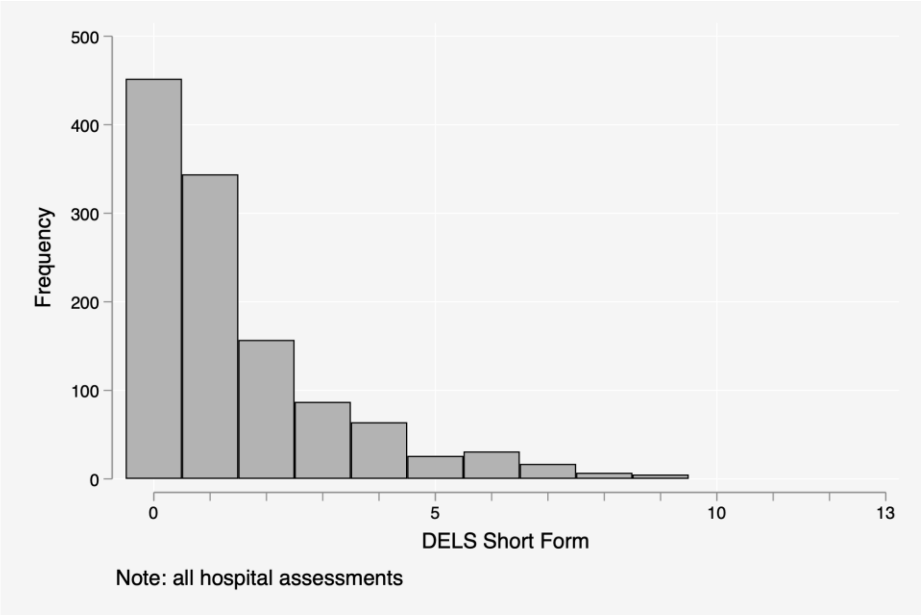

© 2022 Vasunilashorn SM et al. *JAMA Network Open.*

eFigure 2B
Histogram of DELS-LF among 1,190 assessments conducted in 352 patients during hospitalization

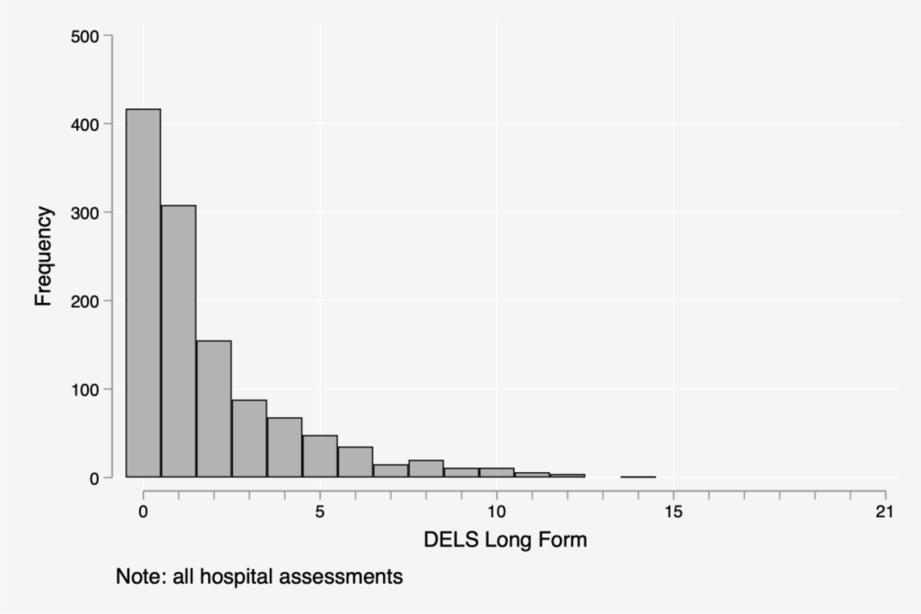

© 2022 Vasunilashorn SM et al. *JAMA Network Open.*

eReferences

Cohen, J. (1988). Statistical power analysis for the behavioral sciences. Hillsdale, New Jersey: Lawrence Erlbaum Associates.

Gelman, A. (2008). Scaling regression inputs by dividing by two standard deviations. Statistics in Medicine, 27(15), 2865.

Gou RY, Hshieh TT, Marcantonio ER, et al. (2021). One-Year Medicare Costs Associated with Delirium in Older Patients Undergoing Major Elective Surgery. JAMA Surg, 156(5), 430–442.

Hshieh, T. T., Fong, T. G., Schmitt, E. M., Marcantonio, E. R., D'Aquila, M. L., Gallagher, J., . . . for the, BASIL Study Group. (2019). The Better Assessment of Illness Study for Delirium Severity: Study Design, Procedures, and Cohort Description. Gerontology, 65(1), 20-29.

McDonald, R. (1999). Test Theory: A Unified Treatment. Mahwah, NJ: Erlbaum.
Nunnally, J. C., & Bernstein, I. H. (1994). Psychometric theory (3rd ed.). New York: McGraw-Hill College Division.

Seaman, S. R., & White, I. R. (2013). Review of inverse probability weighting for dealing with missing data. Statistical Methods in Medical Research, 22(3), 278-295.

Vasunilashorn, S. M., Schulman-Green, D., Tommet, D., Fong, T. G., Hshieh, T. T., Marcantonio, E. R., Metzger, E. D., Schmitt, E. M., Tabloski, P. A., Travison, T. G., Gou, Y., Helfand, B., Inouye, S. K., Jones, R. N., & the Basil Study Team. (2020). New Delirium Severity Indicators: Generation and Internal Validation in the Better Assessment of Illness (BASIL) Study. Dementia and Geriatric Cognitive Disorders, 49(1), 77-90.

© 2022 Vasunilashorn SM et al. *JAMA Network Open.*
