# Supplementary material for: Psychometric Properties of a Delirium Severity Score for Older Adults and Association With Hospital and Posthospital Outcomes
Source: JAMA Netw Open. 2022 Mar 31;5(3):e226129. doi: 10.1001/jamanetworkopen.2022.6129 (PMC8972033; doi:10.1001/jamanetworkopen.2022.6129)
Supplement: Supplement 2. — BASIL Study Team [file jamanetwopen-e226129-s002.pdf]

Supplemental Online Content: Nonauthor Collaborators

*Indicates required information. Only first name, last name, and suffix will appear in PubMed.

*Group Name(s): BASIL Study Team							
***First Name and Middle Initial(s)**	***Last Name**	***Suffix (eg, Jr, III)**	Academic Degrees	Institution	Location (city, state/province, country)	Role or Contribution, eg, chair, principal investigator	Group (if more than 1 Group listed in the byline) and/or Subgroup (eg, Steering Committee)
Dena	Schulman-Green		PhD	Yale University	New Haven, CT, USA	Co-Investigator	n/a
Tatiana	Abrantes		BS	Hebrew SeniorLife	Boston, MA, USA	Field Team	n/a
Brett	Armstrong		MPH	Beth Israel Deaconess Medical Center	Boston, MA, USA	Field Team	n/a
Sylvia	Bertrand		BS	Hebrew SeniorLife	Boston, MA, USA	Field Team	n/a
Angelee	Butters		MA	Beth Israel Deaconess Medical Center	Boston, MA, USA	Field Team	n/a
Madeline	D'Aquila		BS	Hebrew SeniorLife	Boston, MA, USA	Field Team	n/a
Jacqueline	Gallagher		MS	Beth Israel Deaconess Medical Center	Boston, MA, USA	Field Team	n/a
Jennifer	Kettell		BS	Hebrew SeniorLife	Boston, MA, USA	Field Team	n/a
Jacqueline	Nee		BA	Hebrew SeniorLife	Boston, MA, USA	Field Team	n/a
Katelyn	Parisi		BA	Hebrew SeniorLife	Boston, MA, USA	Field Team	n/a
Margaret	Vella		BS	Hebrew SeniorLife	Boston, MA, USA	Field Team	n/a
Guoquan	Xu		MD, PhD	Hebrew SeniorLife	Boston, MA, USA	Field Team	n/a
Lauren	Weiner		MA	Beth Israel Deaconess Medical Center	Boston, MA, USA	Field Team	n/a
Douglas	Tommet		MPH	Brown University	Providence, RI, USA	Data Management and Statistical Analysis Team	n/a
Caroline	Bader		MD, PhD	Beth Israel Deaconess Medical Center; McLean Hospital	Boston, MA, USA; Belmont, MA, USA	Collaborator	n/a
Kristen	Erickson			Hebrew SeniorLife	Boston, MA, USA	Collaborator	n/a
Sunil	Swami		PhD	Hebrew SeniorLife	Boston, MA, USA	Collaborator	n/a
Charles H.	Brown		MD	Johns Hopkins University	Baltimore, MD, USA	Expert Review Panel	n/a
Sevdenur	Cizginer		MD	Brown University	Providence, RI, USA	Expert Review Panel	n/a
Diane	Clark		PT, DScPT, MBA	University of Alabama	Birmingham, AL, USA	Expert Review Panel	n/a

Supplemental Online Content: Nonauthor Collaborators

*Indicates required information. Only first name, last name, and suffix will appear in PubMed.

***First Name and Middle Initial(s)**	***Last Name**	***Suffix (eg, Jr, III)**	Academic Degrees	Institution	Location (city, state/province, country)	Role or Contribution, eg, chair, principal investigator	Group (if more than 1 Group listed in the byline) and/or Subgroup (eg, Steering Committee)
Joseph H.	Flaherty		MD	St. Louis University	St Louis, MO, USA	Expert Review Panel	n/a
Anne	Gleason		BS	Hebrew SeniorLife	Boston, MA, USA	Expert Review Panel	n/a
Ann M.	Kolanowski		PhD, RN	Penn State	University Park, PA, USA	Expert Review Panel	n/a
Karen J.	Neufeld		MD, MPH	Johns Hopkins University	Baltimore, MD, USA	Expert Review Panel	n/a
Margaret G.	O'Connor		PhD	Beth Israel Deaconess Medical Center	Boston, MA, USA	Expert Review Panel	n/a
Margaret A.	Pisani		MD, MPH	Yale University	New Haven, CT, USA	Expert Review Panel	n/a
Thomas	Robinson		MD	University of Colorado	Aurora, CO, USA	Expert Review Panel	n/a
Joe	Verghese		MB, BS	Albert Einstein College of Medicine	Bronx, NY, USA	Expert Review Panel	n/a
Heidi	Wald		MD, MPH	University of Colorado	Aurora, CO, USA	Expert Review Panel	n/a
Sharon M.	Gordon		PsyD	Vanderbilt University	Nashville, TN, USA	Expert Review Panel	n/a
